# Genome-wide gain-of-function screening characterized lncRNA regulators for tumor immune response

**DOI:** 10.1126/sciadv.add0005

**Published:** 2022-12-07

**Authors:** Yifei Wang, Yueshan Zhao, Weiwei Guo, Ghanshyam Singh Yadav, Chetana Bhaskarla, Zehua Wang, Xiaofei Wang, Sihan Li, Yue Wang, Yuang Chen, Dhamotharan Pattarayan, Wen Xie, Song Li, Binfeng Lu, Udai S. Kammula, Min Zhang, Da Yang

**Affiliations:** ^1^ Center for Pharmacogenetics, Department of Pharmaceutical Sciences, University of Pittsburgh, Pittsburgh, PA 15261, USA.; ^2^ UPMC Hillman Cancer Institute, University of Pittsburgh, Pittsburgh, PA 15261, USA.; ^3^ Department of Immunology, University of Pittsburgh, Pittsburgh, PA 15261, USA.; ^4^ Division of Surgical Oncology, Department of Surgery, University of Pittsburgh School of Medicine, University of Pittsburgh Cancer Institute, Pittsburgh, PA 15213, USA.; ^5^ Department of Computational and Systems Biology, University of Pittsburgh, Pittsburgh, PA 15261, USA.

## Abstract

The majority of lncRNAs’ roles in tumor immunology remain elusive. This project performed a CRISPR activation screening of 9744 lncRNAs in melanoma cells cocultured with human CD8^+^ T cells. We identified 16 lncRNAs potentially regulating tumor immune response. Further integrative analysis using tumor immunogenomics data revealed that *IL10RB-DT* and *LINC01198* are significantly correlated with tumor immune response and survival in melanoma and breast cancer. Specifically, *IL10RB-DT* suppresses CD8^+^ T cells activation via inhibiting IFN-γ–JAK–STAT1 signaling and antigen presentation in melanoma and breast cancer cells. On the other hand, *LINC01198*’s up-regulation sensitizes the killing of tumor cells by CD8^+^ T cells. Mechanistically, *LINC01198* interacts and activates NF-κB component p65 to trigger the type I and type II interferon responses in melanoma and breast cancer cells. Our study systematically characterized novel lncRNAs involved in tumor immune response.

## Teaser

This project used a high-throughput gain-of-function screening to identify non-coding genes that regulate cancer immune response.

## INTRODUCTION

Tumor immune surveillance is a critical process in inhibiting tumorigenesis. The immune system is capable of identifying and eliminating potentially cancerous cells (*1*, *2*). During this process, effector T cells play a vital role (*3*). CD8^+^ T cells are activated after T cell receptors (TCRs) recognize tumor neoantigens presented on major histocompatibility complex class I (MHC-I) molecule, resulting in the killing of cancer cells (*4*–*6*). By stimulating the patients’ own immune systems to selectively kill cancer cells, immunotherapy can substantially improve the treatment outcomes for patients with cancer (*7*). Multiple immune checkpoint inhibitors have been approved because of the enhancement of T cell antitumor response in multiple cancer types (*8*–*10*), either as monotherapy or combination therapy (*11*–*13*). Nevertheless, the low response rate (*14*) and undesired side effects (*15*) for some patients suggest that the underlying regulation of tumor immune response has not been fully illustrated.

Accumulating evidence has shown that long noncoding RNAs (lncRNAs) play critical roles in the development of various diseases such as cancer (*16*). Functionally, lncRNAs regulate various cellular functions such as cell differentiation, cell proliferation, and the activation of divergent types of immune cells (*17*, *18*). For instance, lncRNA *ITPRIP-1* and *lnc-Lsm3b* are shown to be involved in the innate immune response to viral infection (*19*, *20*). *Lnc-DC* is critical to dendritic cell (DC) differentiation by interacting with transcription factor signal transducer and activator of transcription 3 (STAT3) (*21*). LncRNA *NKILA* regulates T cell sensitivity to immunological elimination by interacting with nuclear factor κB (NF-κB) (*22*). Our previous analysis characterized an onco-lncRNA, *EPIC1*, which suppresses tumor cell antigen presentation and leads to the resistance of anti-programmed cell death protein 1 (PD-1) treatment in breast cancer (*23*). Although these findings have demonstrated that some lncRNAs could modulate the human immune response in the tumor microenvironment, most lncRNAs’ function and their roles in tumor immunity remain unknown.

High-throughput CRISPR screenings have enabled functional characterization of novel genes involved in the regulation of cancer development (*24*), metastasis (*25*), and drug responses (*26*, *27*). Some loss-of-function CRISPR screening studies have identified several essential lncRNAs (*28*, *29*). However, lncRNA genes are generally expressed at a very low basal level (*30*). The depletion screens on the already low-expressed lncRNAs decrease the power of screening (*31*). Moreover, lncRNAs are not sensitive to missense and frameshift mutations and therefore are difficult to be functionally knocked out by the traditional CRISPR-Cas9 system. These characteristics greatly limit the sensitivity and specificity of lncRNA loss-of-function screening. A recently developed CRISPR activation (CRISPR-SAM) (*32*) technology can activate gene expression by targeting their promoter region. Moreover, the activated transcript will go over similar endogenous posttranscriptional modification and lead to a relatively “physiological” level of overexpression. The CRISPR-SAM technology provides a unique opportunity to systematically characterize novel lncRNA regulators in tumor immune response.

In this study, we conduct a genome-wide gain-of-function CRISPR-SAM screening targeting 9744 ncRNAs using an ex vivo CD8^+^ T cell and tumor cell coculture assay. By further integrating with tumor genomics data of 33 cancer types from The Cancer Genome Atlas (TCGA) database, our screen found two candidate lncRNA genes (*IL10RB-DT* and *LINC01198*) that can potentially regulate tumor cell resistance or sensitivity to T cell cytotoxicity in multiple cancer types. Mechanistically, *IL10RB-DT* regulates tumor necrosis factor–α (TNF-α), interferon-α (IFN-α), and IFN-γ signaling pathways. *LINC01198* regulates tumor inflammation signaling pathway through activation and interaction with NF-κB component p65. By systematically screening for lncRNAs involved in tumor immune response, our study identified potential therapeutic targets and markers of cancer immunotherapy.

## RESULTS

### Genome-wide CRISPRa screen identifies regulatory lncRNAs for tumor cell response to CD8^+^ T cell–mediated cytotoxicity

**Fig. 1.**
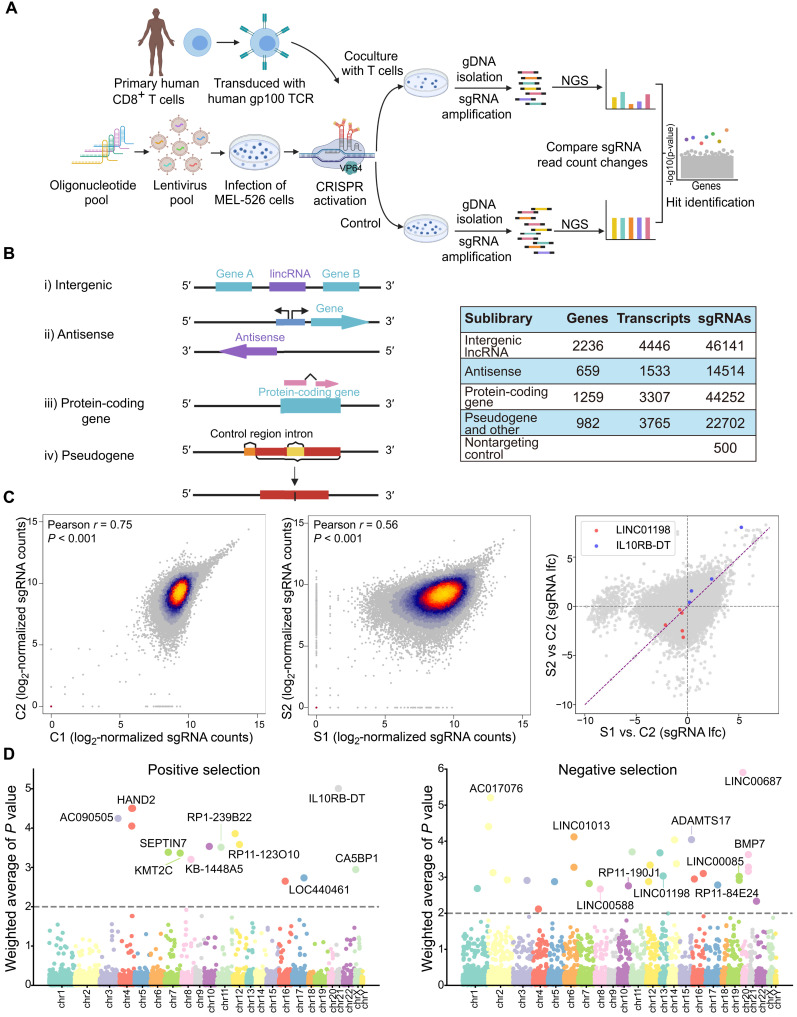
Genome-wide CRISPRa screen identifies regulatory lncRNAs for tumor cell response to CD8^+^ T cell–mediated cytotoxicity. (**A**) Overview of CRISPRa screen in a tumor cell and T cell coculture system. (**B**) Different types of transcripts can be targeted in the sgRNA library. Left: Relationships between lncRNAs and PCGs in the genome. Right: The number of transcripts that are targeted in the library. (**C**) Left and middle: Scatter plots for sgRNA read counts between controls (C1 and C2) and cocultured replicates (S1 and S2), respectively. Right: Scatter plot for log-transformed fold change of sgRNAs in the two comparisons (S1 versus C2 and S2 versus C2, Pearson *r* = 0.26, *P* < 0.05). (**D**) Weighted average RIGER analysis *P* values for negative and positive selections.

To characterize lncRNAs that regulate the tumor cell interaction with cytotoxic T cells, we performed a genome-wide lncRNA activation screening using our established primary human CD8^+^ T cell and melanoma cell coculture system (*33*). Briefly, the human primary CD8^+^ T cells were transduced with a specific TCR-recognized human gp100 peptide antigen, which can be presented in an human leukocyte antigen A (HLA-A*02)–specific manner of human melanoma cell line MEL-526 . We then established the CRISPR-SAM system in MEL-526 cells and transduced the cells with a genome-scale CRISPRa library (*32*) including 96,458 single-guide RNAs (sgRNAs) designed to target lncRNA transcriptional start site (TSS) (Fig. 1A and see Materials and Methods). On the basis of the GENCODE v19, the library contained 500 nontargeting control sgRNAs and sgRNAs targeting 9744 transcripts of noncoding genes, including 4446 intergenic lncRNAs and 1533 antisense lncRNAs (Fig. 1B). In total, we had five independent samples with one original library control (plasmid) and two sgRNA-transduced control samples with different cultured periods (C1 and C2; Fig. 1A). We have included two samples as biological replicates that have cocultured with human gp100 TCR-transduced CD8^+^ T cell for 4 hours (S1 and S2) (Fig. 1A and fig. S1A).

After analyzing the sequence result, sgRNA abundance showed a significant positive correlation between two control samples (Pearson *r* = 0.75, *P* < 0.001) and two cocultured replicates (Pearson *r* = 0.56, *P* < 0.001; Fig. 1C). After coculture with CD8^+^ T cells, the sgRNA abundance showed a more skewed distribution with more enriched or depleted sgRNAs compared with controls (fig. S1B), suggesting that coculturing with CD8^+^ T cells exerted selective pressure on the cancer cells. The top enriched target genes for both positive (enriched after coculture) and negative (depleted after coculture) selected genes were characterized by RNAi Gene Enrichment Ranking (RIGER; Fig. 1D) in individual biological replicates compared to the noncoculturing control (C2). Genes that were significantly enriched/depleted in both replicates (see Materials and Methods for details) were identified for further analysis (Fig. 1D and fig. S1C). For each identified gene, sgRNAs (table S1) were consistently enriched or depleted after being cocultured with CD8^+^ T cells compared with control (fig. S1D). To increase the reliability of positive and negative selections for further functional validation, we also performed MAGeCK (Model-based Analysis of Genome-wide CRISPR-Cas9 Knockout) (*34*) analysis (see Materials and Methods). The combined RIGER and MAGeCK analysis identified 16 candidate genes that may regulate cancer cell response to antigen-mediated T cell cytotoxicity (Fig. 1D and table S2). We have also shown the genomics localization of the top 10 targets from both positive and negative selections and the nearby genes (fig. S1, E and F).

### CRISPRa screen recapitulates protein-coding gene ULK1’s repression of antitumor immunity

Because some protein-coding genes (PCGs) share TSS with the lncRNAs, the CRISPRa lncRNA library can potentially activate 1259 PCGs (Fig. 1B and Materials and Methods). In this regard, we first determine whether the CRISPRa screening can identify some established PCG immune modulators. Among these negative-selected PCGs are F-box only protein 11 (FBXO11) (*35*) and F-box/WD repeat-containing protein (FBXW7) (*36*), which have been previously demonstrated to be involved in cancer immunology.

**Fig. 2.**
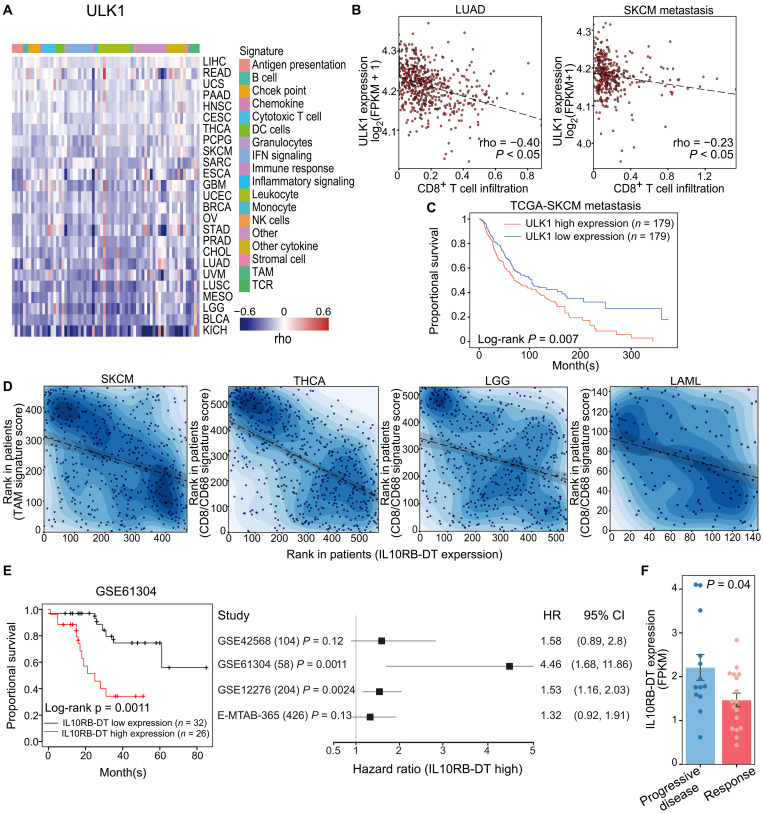
CRISPRa screen characterizes PCG *ULK1* and lncRNA *IL10RB-DT* as tumor immune response suppressors. (**A**) *ULK1* showed a significant negative expression correlation with 68 immune signatures in multiple TCGA cancer types. NK, natural killer. (**B**) *ULK1*’s expression negatively correlated with CD8^+^ T cell infiltration in Lung adenocarcinoma (LUAD) and metastatic Skin Cutaneous Melanoma (SKCM) patients. (**C**) TCGA metastatic melanoma patients with high ULK1 expression showed poor overall survival. (**D**) *IL10RB-DT*’s expression negatively correlated with TAM/CD8 signature in TCGA SKCM, Thyroid carcinoma (THCA), Acute Myeloid Leukemia (LAML), and Brain Lower Grade Glioma (LGG) patients. (**E**) Forest plot of hazard ratios (HR) for overall survival of patients with breast cancer according to *IL10RB-DT*’s expression in four independent cohorts. *IL10RB-DT*’s expression is measured by Affymetrix probe 230632_at in the HG-U133_Plus_2 platform. (**F**) The anti–PD-1 responders showed lower IL10RB-DT expression than nonresponders in patients with melanoma (*P* = 0.04).

In addition to the negative-selected PCGs, we also identified positively selected PCGs, including unc-51 like autophagy activating kinase 1 (ULK1), which may lead to resistance to T cell cytotoxicity. Among the 10 sgRNAs targeting the promoter region of ULK1, six showed significant enrichment in both replicates (fig. S2A). Meanwhile, ULK1’s expression showed a significant negative correlation with the activity and infiltration of CD8^+^ cytotoxic T cells in 12 cancer types including lung cancer and melanoma in the TCGA dataset (Fig. 2B and fig. S2B). Consistent with its role in T cell cytotoxicity resistance observed in our CRISPRa screening, ULK1’s expression was correlated with a poor survival rate in patients with metastatic melanoma (Fig. 2C). These results suggested that ULK1 played an immune suppressive role in multiple cancer types. ULK1 was recently identified as an oncogene that suppressed antitumor immune response in lung cancer (*37*). Mechanistically, ULK1 inhibited immunoproteasome activity by inducing autophagic flux. Pharmacological repression of ULK1 led to a synergized therapeutic effect with anti–PD-1 treatment (*37*). The confirmation of ULK1’s immune suppressive function suggested that our CRISPRa screening used the coculture system of CD8^+^ T cells, and tumor cells could successfully identify the established immune regulator.

### Integrative analyses of cancer genomics and CRISPRa screen data identify *IL10RB-DT* as a novel lncRNA-suppressing tumor immune response

The recapitulation of ULK1’s immune-modulatory role in the gain-of-function screening motivated us to investigate the potential immune-regulating lncRNAs from our screening result. Among the eight positive-selected lncRNAs, *IL10RB-DT* caught our attention. Fifty percent of sgRNAs designed to target *IL10RB-DT* TSS were significantly enriched in MEL-526 cells after coculturing with gp100^+^ CD8^+^ T cells (fig. S2C). Consistent with its potential immune inhibitory effect revealed by CRISPRa screening, *IL10RB-DT* showed higher expression levels in tumor tissues including melanoma, pancreatic adenocarcinoma, and kidney carcinoma (fig. S2D). In addition, *IL10RB-DT* expression was negatively correlated with tumor-associated macrophage (TAM) signature in 472 patients with melanoma (rho = −0.3, *P* < 0.05; Fig. 2D) and CD8/CD68 signature in 568 patients with thyroid cancer (rho = −0.53, *P* < 0.05), 151 patients with acute myeloid leukemia (rho = −0.28, *P* < 0.05), and 529 patients with low-grade glioma (rho = −0.29, *P* < 0.05). *IL10RB-DT* expression was also significantly correlated with poor survival in patients with breast cancer (Fig. 2E). Further analysis of the transcriptomic data and the anti–PD-1 therapy response in a cohort of 30 patients with metastatic melanoma (*38*) revealed that tumors with higher *IL10RB-DT* expression are resistant to anti–PD-1 treatment (Fig. 2F). These results suggested that *IL10RB-DT* played an immune-suppressing role in multiple cancer types and could be a potential biomarker for immunotherapy treatment.

**Fig. 3.**
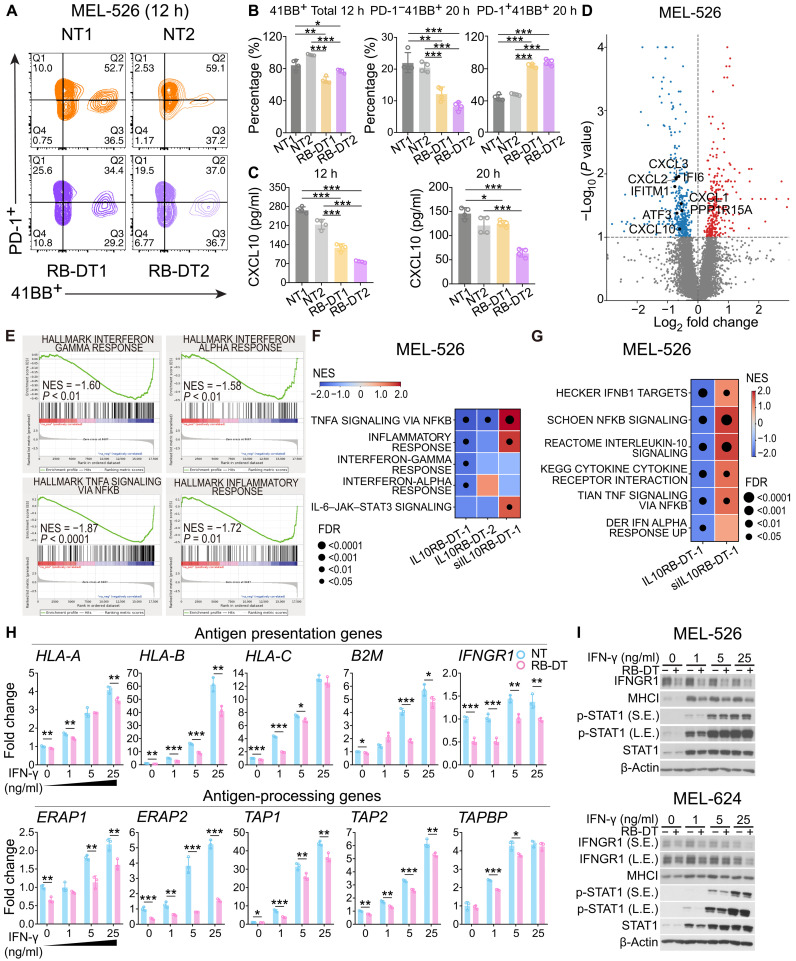
*IL10RB-DT* suppresses tumor antigen presentation via IFN-γ–JAK–STAT1 signaling. (**A**) Representative fluorescence-activated cell sorting (FACS) analysis panel of gp100 TCR-transduced CD8^+^ T cells cocultured with MEL-526 cells transfected with either nontargeting sgRNA (NT) control or single sgRNA–activated *IL10RB-DT* (RB-DT) with *E*:*T* ratio of 0.5:1 for 12 hours. (**B**) Quantification of gp100 TCR-transduced CD8^+^ T activation level for 12 and 20 hours of coculture. The data shown are representative of three independent experiments (mean ± SD, two-tailed *t* test, unpaired). (**C**) ELISA detection of CXCL10 secretion level from 12-hour (left) and 20-hour (right) coculture supernatant. The data shown are representative of three independent experiments (mean ± SD, two-tailed *t* test, unpaired). (**D**) Volcano plot for differential expression between MEL-526 cells with *IL10RB-DT* activation and control sample (NT). (**E**) Genes in immune-related pathways showed decreased expression in *IL10RB-DT*–activated MEL-526 cells. (**F** and **G**) Cancer hallmarks (F) and curated gene sets (G) are enriched with up- or down-regulated genes in MEL-526 cells with *IL10RB-DT* activations compared to the control sample using GSEA analysis. The normalized enrichment score (NES) and FDR for each pathway are shown. (**H**) RT-PCR result showing the mRNA expression level of antigen presentation and processing genes in MEL-526 control (NT) and *IL10RB-DT*–activated cells (RB-DT) treated with IFN-γ (0, 1, 5, and 25 ng/ml) for 24 hours (*n* = 3 independent experiments, mean ± SD, two-tailed *t* test, unpaired). (**I**) Representative Western blot of IFNGR1, MHCI, and p-STAT1 in MEL-526 (top) and MEL-624 (bottom) control (−RB-DT) and *IL10RB-DT*–activated (+RB-DT) cells treated with IFN-γ (0, 1, 5, and 25 ng/ml) for 24 hours. Data are representative of three independent experiments. Blots are cropped, and original images can be found in the source data. Statistical significance: *P < 0.05, **P < 0.01, and ***P < 0.001.

To validate *IL10RB-DT*’*s* immune inhibitory effect, we transfected CRISPR-SAM–MEL-526 and human breast cancer cell line MCF-7 with single sgRNAs individually. Compared with control nontarget sgRNAs (NT1 and NT2), two sgRNAs targeting *IL10RB-DT*’s TSS (IL10RB-DT-1 and IL10RB-DT-2) could increase endogenous *IL10RB-DT* expression levels to more than 15- and 12-fold, respectively. In contrast, the PCG *IL10RB* was not activated by sgRNAs, suggesting that the designed sgRNAs can specifically target IL10RB-DT (fig. S2E). We then cocultured the single-sgRNA–activated *IL10RB-DT*–MEL-526 cells and MEL-526 control cells with human gp100^+^ CD8^+^ T cells for different time points and determined their ability to activate the T cells (Fig. 3A). We found that *IL10RB-DT*–activated cells led to a significant decrease in CD8^+^ T cell activation level (41BB^+^) compared with control cells after 6 hours (fig. S3A) and 12 hours (Fig. 3B and fig. S3C) of coculture. We also observed that IFN-γ and CXCL10 secretion were significantly depleted in multiple time points by *IL10RB-DT* activation (Fig. 3C and fig. S3B). In addition, coculturing with *IL10RB-DT*–activated cells significantly induced CD8^+^ T cell’s PD-1 expression level (Fig. 3B) in a longer time point. These observations demonstrated that *IL10RB-DT*’s activation in the tumor cells suppressed CD8^+^ T cell’s activation and induced its exhaustion (*39*).

### *IL10RB-DT* suppresses tumor antigen presentation via IFN-γ–Janus kinase–STAT1 signaling

To determine how *IL10RB-DT* inhibits immune response, we performed RNA sequencing (RNA-seq) of *IL10RB-DT*–activated and small interfering RNA (siRNA) knockdown MEL-526 cells. RNA-seq analyses identified 178 up-regulated and 250 down-regulated genes in MEL-526 *IL10RB-DT* activation samples (Fig. 3D and table S3). The gene set enrichment analysis (GSEA) analyses revealed that immune-related signaling, such as IFN-γ response showed significant down-regulation in *IL10RB-DT*–activated cells (Fig. 3, E to G), which was in accordance with our observation in the coculture assay described above. In addition, several other immune pathways such as INFLAMMATORY and interleukin-6 (IL-6)–Janus kinase (JAK)–STAT3 signaling (Fig. 3, E and F, and fig. S3D) were also suppressed after *IL10RB-DT*’s activation. Compared to the control cells, the top-ranked immune-related genes changed in *IL10RB-DT*–activated and knockdown cells including IFN-stimulated gene 15 (ISG15), C-C Motif Chemokine Ligand 2 (CCL2), and CXCL10 (fig. S3E) further demonstrated *IL10RB-DT*’s regulation of these pathways.

The observations that *IL10RB-DT* activation suppresses IFN-γ signaling in cancer cells and decreases CD8^+^ T cell activation led us to hypothesize that *IL10RB-DT* might regulate IFN-γ and antigen presentation and processing pathways. These two pathways are known to cooperate in the regulation of the adaptive immune system and are directly involved in CD8^+^ T cell recognition of tumor cells (*40*). To test this hypothesis, we first treated *IL10RB-DT*–activated and non-targeting control (NT) MEL-526 cells with IFN-γ and found that the mRNA level of antigen presentation and processing genes and IFNGR1 (IFN-γ receptor 1) were down-regulated in *IL10RB-DT*–activated cells in a dosage-dependent manner (Fig. 3H). To determine whether *IL10BR-DT*’s regulation effect can be generalized to other cell lines and cancer types, we activated *IL10RB-DT*’s expression by a single sgRNA in the melanoma cell line MEL-624 and breast cancer cell line MCF-7. In both cells (fig. S3, F and G), *IL10RB-DT* activation inhibited the transcription level of MHC-I and antigen-processing genes. Moreover, the STAT1 phosphorylation and protein expression of IFNGR1 and MHC-I can be suppressed by *IL10RB-DT* in both MEL-526 and MEL-624 cells (Fig. 3I). On the other hand, *IL10RB-DT* knockdown (fig. S3H) induced the expression of antigen presentation and processing genes in both MEL-526 (fig S3I) and MCF-7 (fig. S3J) cells. These observations suggested that *IL10RB-DT* suppressed tumor antigen presentation through IFN-γ–JAK–STAT1 signaling.

### LncRNA *LINC01198* promotes CD8^+^ T cell–mediated immune response in melanoma and breast cancer

**Fig. 4.**
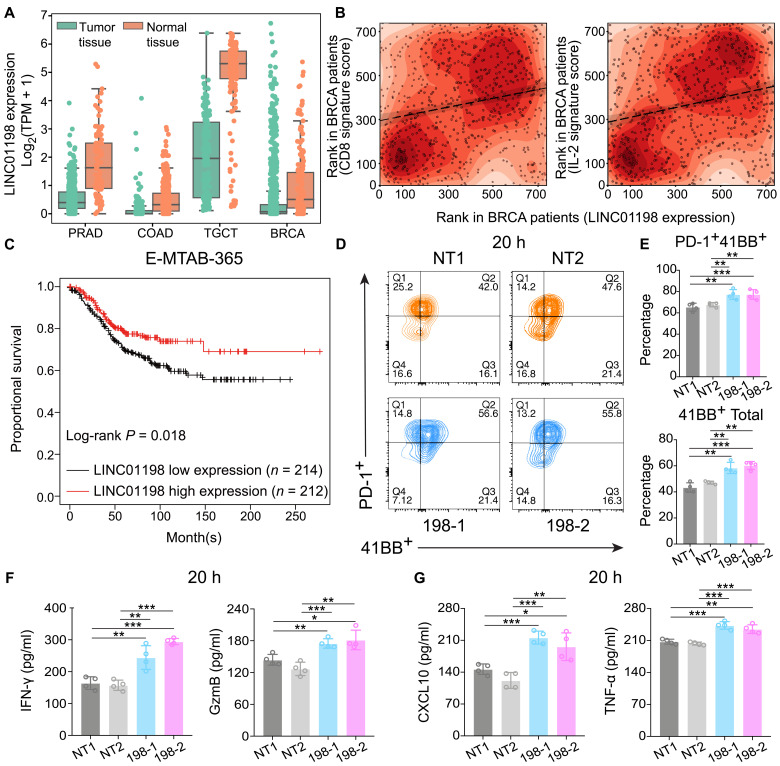
LncRNA *LINC01198* promotes CD8^+^ T cell–mediated immune response in melanoma and breast cancerABCDEF G (**A**) *LINC01198* demonstrated low expression in TCGA Prostate adenocarcinoma (PRAD) (*P* = 0.0016), Colon adenocarcinoma (COAD) (*P* = 3 × 10^−23^), Testicular Germ Cell Tumors (TGCT) (*P* = 3 × 10^−65^), and Breast invasive carcinoma (BRCA) (*P* = 1.9 × 10^−21^) samples compared with GTEx normal samples. (**B**) *LINC01198*’s expression positively correlated with CD8 (left) and IL-2 (right) signature score in TCGA BRCA patients. (**C**) Patients with breast cancer having high *LINC01198* expression showed favorable overall survival. *LINC01198*’s expression is measured by Affymetrix probe 1553614_a_at in the HG-U133_Plus_2 platform. (**D**) Representative FACS analysis panel of gp100 TCR-transduced CD8^+^ T cells cocultured with MEL-526 cells transfected with either nontargeting sgRNA (NT) control or single sgRNA activated *LINC01198* (198) with *E*:*T* ratio of 0.5:1 for 20 hours. (**E**) Quantification of gp100 TCR-transduced CD8^+^ T activation level of (D). The data shown represent one of the three independent experiments with similar results (mean ± SD, two-tailed *t* test, unpaired). (**F** and **G**) IFN-γ, Granzymb (GzmB) (F), and CXCL10, TNF-α (G) secretion level from 20-hour coculture supernatant detected by ELISA. The data shown represent one of three independent experiments with similar results (mean ± SD, two-tailed *t* test, unpaired). Statistical significance: *P < 0.05, **P < 0.01, and ***P < 0.001.

In addition to immune-resistant lncRNAs, the CRISPRa screening also identified eight negative-selected lncRNAs promoting CD8^+^ T cell cytotoxicity (see Fig. 1). Among them, *LINC01198* is one of the top negative-selected lncRNAs. Six of nine sgRNAs that target *LINC01198*’s TSS were significantly depleted (*P* < 0.05) in MEL-526 cells after coculturing with gp100^+^ CD8^+^ T cells (fig. S4A). Under physiological conditions, *LINC01198* has low expression levels in immune-related tissues such as blood and spleen (fig. S4B). Compared to normal samples, *LINC01198* was significantly suppressed in multiple cancer types including breast cancer, colon adenocarcinoma, prostate cancer, and testicular germ cell tumor (Fig. 4A). Patients with breast cancer having higher *LINC01198*’s expression tended to have a higher immune response indicated by higher CD8, IL-2, IL-8, and IL-12 immune signature scores (rho = 0.309, 0.374, 0.555, and 0.359, respectively; *P* < 0.05; Fig. 4B and fig. S4C; see Materials and Methods) and significantly longer overall survival (Fig. 4C).

To validate *LINC01198*’s regulation of tumor immunity, we stably expressed two different sgRNAs targeting *LINC01198*’s TSS in MEL-526 and estrogen receptor-positive (ER^+^) breast cancer cell line MCF-7 (fig. S4D). The up-regulation level of *LINC01198* by sgRNAs could reach more than 13-fold in MEL-526 cells and more than 35-fold compared with NT in MCF-7 cells. Coculturing the human CD8^+^ T cells with MEL-526 *LINC01198*–activated cells revealed a significant promotion of CD8^+^ T cells’ activation level at two time points (Fig. 4, D and E, and fig. S4, E, and F). Furthermore, we observed that *LINC01198*’s activation significantly stimulated the secretion of granzyme B (GzmB), IFN-γ, CXCL10, and TNF-α from multiple time points (Fig. 4, F and G, and fig. S4, G and H). These findings illustrated that *LINC01198* in the tumor cells could enhance the cytotoxicity of CD8^+^ T cells and might further facilitate T cells targeting tumor cells by regulating the secretion of cytokines.

### *LINC01198* regulates IFN signaling pathways

**Fig. 5.**
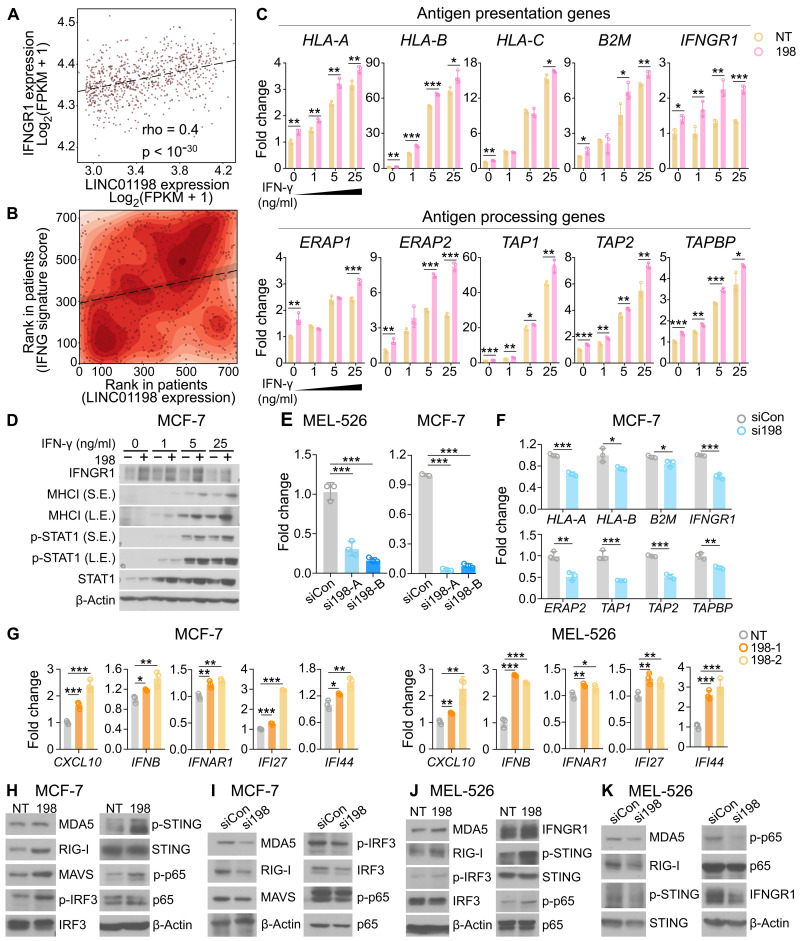
*LINC01198* regulates IFN signaling pathways. (**A** and **B**) *LINC01198*’s expression is positively correlated with IFNGR1 expression (A) and IFNG signature score (B) in TCGA BRCA patients (rho = 0.31, *P* < 0.05). (**C**) RT-PCR result showing the mRNA expression level of IFN-γ and its related genes in MEL-526 control (NT) and *LINC01198*-activated (198) cells treated with IFN-γ (0, 1, 5, and 25 ng/ml) for 24 hours (*n* = 3 independent experiments, mean ± SD, two-tailed *t* test, unpaired). (**D**) Representative Western blot of IFNGR1, MHCI, and p-STAT1 in MCF-7 control (−198) and *LINC01198* activated cells (+198) treated with IFN-γ (0, 1, 5, and 25 ng/ml) for 24 hours. Data are representative of three independent experiments. Blots are cropped, and original images can be found in the source data. (**E** and **F**) RT-PCR result showing the knockdown efficiency of *LINC01198* by siRNA treatment in MEL-526 and MCF-7 cells (E) and IFN-γ–related signaling expression level after *LINC01198* or control siRNA treatment in MCF-7 cells (F) (*n* = 3 independent experiments, mean ± SD, two-tailed *t* test, unpaired). (**G**) RT-PCR result showing the mRNA expression level of type I IFN–related genes in control (NT) and *LINC01198*-activated (198) MCF-7 (left) and MEL-526 (right) cells (*n* = 3 independent experiments, mean ± SD, two-tailed *t* test, unpaired). (**H** to **K**) Representative Western blot of type I IFN and antiviral-related protein expression level in MCF-7 (H), MEL-526 (J) control (NT), and *LINC01198*-activated (198) cells; in MCF-7 (I), MEL-526 (K) control, and *LINC01198* siRNA–treated cells. Data are representative of three independent experiments. Blots are cropped, and original images can be found in the source data. Statistical significance: *P < 0.05, **P < 0.01, and ***P < 0.001.

In tumor samples, *LINC01198*’s expression level was positively associated with IFNGR1 expression (Fig. 5A) and IFN-γ signature (Fig. 5B). Combined with the observation that *LINC01198*’s activation in tumor cells increased IFN-γ, GzmB, and type I–related cytokine (i.e., CXCL10 and TNF-α) secretions, we hypothesized that *LINC01198* regulated IFN signaling pathways in cancer cells. In *LINC01198-*activated MEL-526 (Fig. 5C) and MEL-624 (fig. S5A) cells, the expressions of IFNGR1 downstream genes [e.g., transporter associated with antigen processing 1/2 (TAP1/2), endoplasmic reticulum aminopeptidase 1/2 (ERAP1/2), and TAP-associated glycoprotein (TAPBP)] were significantly up-regulated in an IFN-γ–dependent manner. The basal mRNA expression of IFNGR1-related genes (fig. S5, B and C) and the protein expression level of MHC-I and STAT1 phosphorylation (Fig. 5D and fig. S5D) were also elevated by *LINC01198* activation in both MEL-526 and MCF-7 cells. On the other hand, inhibition of *LINC01198* by siRNA (Fig. 5E) showed repressed expression of IFNGR1 downstream genes in both MCF-7 (Fig. 5F) and MEL-526 (fig. S5E) cells.

In addition to the IFN-γ signaling, *LINC01198*-activated MCF-7 and MEL-526 (Fig. 5G and fig.S5F) cells induced the expression of CXCL10, IFN-β, type I IFN receptors, and ISGs. Moreover, knockdown of *LINC01198* by siRNA repressed the transcription level (fig. S5G) of type I IFN pathway–related genes, including type I IFN receptors (IFNAR1/2), type I IFNs, and ISGs in MCF-7 cells. Consistent with its inhibition of type I IFN signaling, antiviral signaling targets such as [melanoma differentiation-associated protein 5 (MDA5), mitochondrial antiviral signaling protein (MAVS), and retinoic acid-inducible gene I (RIG-I)] that are known to cooperate with type I IFNs in innate immunity were also regulated by *LINC01198* at the protein level in MCF-7 (Fig. 5, H and I) and MEL-526 (Fig. 5, J and K) cells. These results suggested that *LINC01198*-mediated type I and type II IFN pathways’ activation might contribute to its immune-promoting function.

### RNA-seq analysis and further mechanism study reveal that *LINC01198* interacts and activates NF-κB component p65

**Fig. 6.**
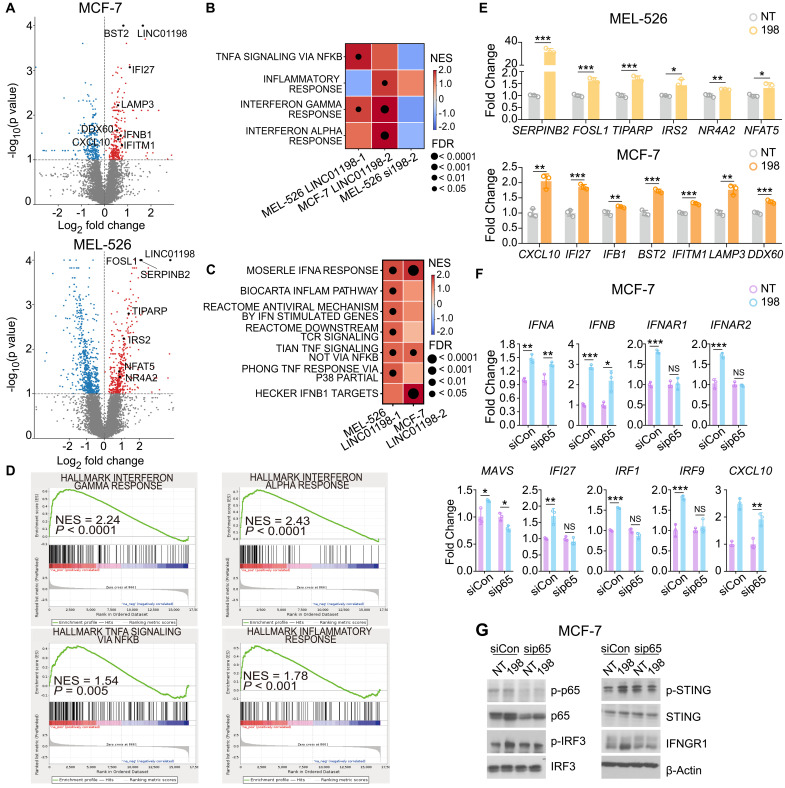
RNA-seq analysis reveals that *LINC01198*’s function is dependent on NF-κB component p65. (**A**) Volcano plot of differential expression between control and *LINC01198* activation samples in MCF-7 (top) and MEL-526 (bottom) cells. (**B** and **C**) Cancer hallmarks (B) and curated gene sets (C) are enriched with up- or down-regulated genes in MCF-7 and MEL-526 cells with *LINC01198* activation/knockdown compared to the control sample using GSEA analysis. The NES and FDR for each pathway were shown. (**D**) Genes in immune-related pathways showed increased expression in *LINC01198-*activated MCF-7 cells compared to the control sample. (**E**) RT-PCR result showing validation of RNA-seq result from (A) in MEL-526 and MCF-7 control (NT) and *LINC01198*-activated (198) cells (*n* = 3 independent experiments, mean ± SD, two-tailed *t* test, unpaired). (**F)** RT-PCR result showing the mRNA expression level of type I IFN–related genes in MCF-7 control (NT) and *LINC01198*-activated (198) cells treated with either control or p65 siRNA. The expression level in *LINC01198*-activated cells were normalized with its own control sample (*n* = 3 independent experiments, mean ± SD, two-tailed *t* test, unpaired). (**G**) Representative Western blot of type I IFN–related and IFNGR1 protein expression level in MCF-7 control (NT) and *LINC01198-*activated (198) cells treated with either control or p65 siRNA. Data are representative of three independent experiments. Blots are cropped, and original images can be found in the source data. Statistical significance: *P < 0.05, **P < 0.01, and ***P < 0.001. NS, not significant.

To further uncover the underlying mechanism of *LINC01198*’s participation in type I and type II IFN pathways, we performed RNA-seq analysis on MCF-7 and MEL-526 cells with *LINC01198*’s activation. The RNA-seq analysis identified 219 induced and 400 depleted genes’ expressions in MEL-526 cells (table S3). Meanwhile, we also identified 93 up-regulated and 63 down-regulated targets in MCF-7 cells (Fig. 6A and table S3). In accordance with our previous observations, genes participated in type I (TIPARP, SERPINB2, IFI27, and LAMP3) and type II (CXCL10, DDX60, and BST2) IFN-related signaling pathways were up-regulated in *LINC01198*-activated (Fig. 6, B and C) MCF-7 (Fig. 6D) and MEL-526 cells (fig. S6A). The *LINC01198-*regulated genes were further validated by the quantitative real-time polymerase chain reaction (qRT-PCR) (Fig. 6E) and showed a high correlation with *LINC01198*’s expression in patients with breast cancer (fig. S6, B to D).

Using the top *LINC01198-*regulated genes, we constructed a *LINC01198* activation score (see Materials and Methods) and evaluated whether it can predict immunotherapy response in 63 patients with cancer who received anti–PD-1 treatment (see Materials and Methods) (*41*, *42*). This analysis revealed that tumors with a higher *LINC01198* signature score showed a trend of better response to anti–PD-1 treatment (fig. S6E). Consistent with its association with the anti–PD-1 response, the *LINC01198* activation signature was positively correlated with a better tumor immune response after treatment, indicating higher MHC-I expression, and more cytotoxic T cell infiltration (fig. S6, F and G).

Further pathway analysis revealed that *LINC01198* can strongly up-regulate NF-κB downstream targets (Fig. 6, B and D). NF-κB is a transcription factor containing two subunits p65 [v-rel avian reticuloendotheliosis viral oncogene homolog A (RELA)]) and p50 and plays a critical role in regulating innate immunity. Triggered by its upstream regulator, such as TNF-α, NF-κB activates a network responsible for complicated biological pathways (*43*). Recently, NF-κB has been found to directly manipulate type I IFN signaling pathway by inducing the expression of IFN-α/β (*44*) and IFN regulatory factors (IRFs), which also could regulate the IFN-γ signaling pathway (*45*). To determine whether *LINC01198*’s function is dependent on p65, we used siRNA to knock down p65 in control (NT) and *LINC01198*-activated MCF-7 cells. This analysis revealed that knockdown of p65 can rescue *LINC01198*’s regulation of type I IFN–related genes, as well as antigen presentation and processing genes, in mRNA and protein levels (Fig. 6, F and G, and fig. S7A). In this regard, we hypothesized that *LINC01198* regulates type I and II IFN pathways through interaction with NF-κB component p65.

**Fig. 7.**
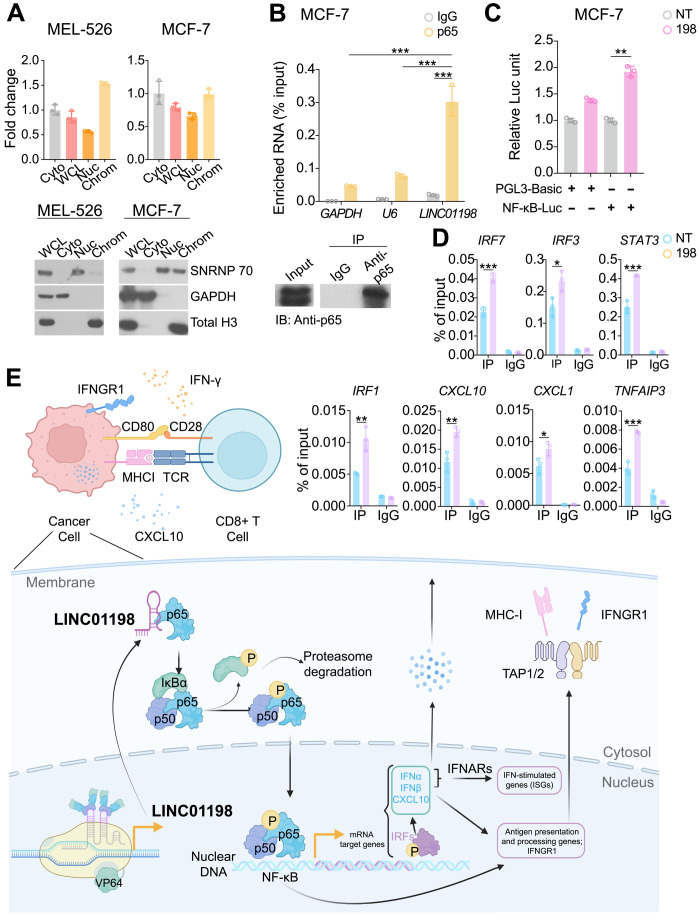
Mechanism study indicates that *LINC01198* interacts and activates p65. (**A**) RT-PCR result of *LINC01198* expression (top) and Western blot (bottom) of fractionation assay in MEL-526 (left) and MCF-7 (right) cells. U1 small nuclear ribonucleoprotein 70 kDa (SNRP70), GAPDH, and total H3 served as a specific nuclear, cytoplasmic, chromatin marker in whole-cell lysates (WCL), cytoplasmic (Cyto), nuclear (Nuc), and chromatin (Chrom) fractionation. Data shown represent one of the three independent experiments with similar results (mean ± SD, two-tailed *t* test, unpaired). Blots are cropped, and original images can be found in the source data. (**B**) RT-PCR result (top) of RIP assay of *LINC01198* enriched by p65 protein in MCF-7 *LINC01198*-activated cells (198). Western blot of p65 IP efficacy is shown (bottom). *GAPDH* and *U6* served as negative controls (mean ± SD, two-tailed *t* test, unpaired). (**C**) Relative luciferase units are shown for reporter assay of either PGL3-Basic plasmid or PGL3 plasmid inserted with 5× NF-κB responsive elements in MCF-7 control (NT) and *LINC01198*-activated (198) cells (*n* = 3 independent experiments, mean ± SD, two-tailed *t* test, unpaired). (**D**) ChIP-qPCR result of p65 occupancy on the promoters of its target genes in MCF-7 control (NT) and *LINC01198*-activated (198) cells (*n* = 3 independent experiments, mean ± SD, two-tailed *t* test, unpaired). (**E**) Proposed model depicting the regulation of immune response by lncRNA *LINC01198* in tumor cells. Statistical significance: *P < 0.05, **P < 0.01, and ***P < 0.001.

The p65 is mainly activated by its upstream regulator such as TNF-α in the cytoplasm (*46*) and then translocated to nuclear to regulate downstream gene expression. Cell fractionation analysis revealed that *LINC01198* is located in both the nucleus and cytoplasm in multiple cell lines (Fig. 7A and fig. S7, B and C), which suggested that it may interact with p65 and be involved in p65 phosphorylation and activation. *LINC01198*’s activation (Fig. 5, H and J) and knockdown (Fig. 5, I and K) can accordingly regulate p65 activation without changing its expression. Further RNA immunoprecipitation (RIP) demonstrated that endogenous p65 protein can enrich *LINC01198* RNA (Fig. 7B). We next sought to determine *LINC01198*’s effect on p65 transcriptional activity using NF-κB luciferase reporter assay. We observed that activation of *LINC01198* significantly promoted NF-κB transcription activity in MCF-7 cells (Fig. 7C). To further validate our hypothesis, we performed a p65 chromatin immunoprecipitation (ChIP)–qPCR analysis on the promoters of *LINC01198-*regulating IFN signaling genes including CXCL10, IRF1, and IRF3. This analysis demonstrated that *LINC01198*’s up-regulation significantly induced p65 occupancy on the promoters of its target genes (Fig. 7D and fig. S7E). Together, these observations suggested that *LINC01198*’s immune-modulatory function relies on p65 (Fig. 7E).

## DISCUSSION

With the function of the vast majority of lncRNAs remaining unknown, the genome-wide CRISPR screen would have been an excellent platform to characterize those novel genes. However, because of lncRNAs’ low expression levels and noncoding nature (*47*), genome-wide functional screening of lncRNA has not gained too much momentum (*30*, *31*). In this study, we have performed a systematic gain-of-function screening and identified novel lncRNAs that play critical roles in the interaction between tumor and cytotoxic T cells. We have shown that our CRISPRa screening could recapitulate the established immune modulator PCG ULK1, although the sgRNA library only activated 1259 PCGs as by-products of lncRNA activation. We functionally characterized novel immune-modulating lncRNAs *IL10RB-DT* and *LINC001198*, which regulate T cell activation by inhibiting the type I/II IFN pathways. Mechanistically, we have shown that lncRNA *LINC01198* promotes immune response through the activation of NF-κB component p65 (Fig. 7E).

Before CRISPRa, most of the lncRNA gain-of-function studies rely on plasmid-based overexpression. These studies usually insert the cDNA sequence of the lncRNAs into the plasmid with the artificial promoter to achieve ectopic overexpression. However, the splicing of lncRNA mRNA is very complicated, which generates much more isoform transcripts than PCGs (*48*). Nowadays, even with tremendous sequencing efforts such as ENCODE, most of the lncRNA cDNA sequence are still poorly elucidated. The gain-of-function study of a single lncRNA gene remains tedious work, let alone genome-wide gain-of-function screening. The invention of CRISPR-SAM technology largely alleviates this problem. Because the overexpressed lncRNA product can go through the endogenous posttranscriptional processing and fold into the appropriate secondary structure. As we have shown in our validation, the single sgRNA target of the TSS of two novel lncRNAs can mimic the physiological up-regulation of lncRNAs, which largely facilitated us to characterize their roles in tumor immune regulation.

One limitation of our study is the design of the lncRNA activation library. The design of the lncRNA activation library needs to consider several important factors including well-annotated TSS sites, sufficient coverage of the promoter region, and the existence of different isoforms generated from the same lncRNA gene. In our study, the lncRNA activation library was designed on the basis of an lncRNA annotation generated a decade ago (*49*). This annotation comprised the most known lncRNAs but did not include many newly identified lncRNAs. In addition to that, the exact TSS sites of most lncRNAs are still not fully characterized. These limitations cause some sgRNAs to ineffectively activate the lncRNAs. Moreover, a small number of the sgRNAs turn out to only target the PCGs (table S1). Our reannotation of the sgRNAs to the most updated GENCODE database only alleviated but did not solve these limitations. In future studies, we will use the most updated bioinformatics tools (*50*–*52*) to design the sgRNA library. After the first stage of genome-wide screening, a carefully designed customized screening to validate and prioritize the hits may be a good strategy to overcome these inherent limitations of lncRNA screening.

Another common caveat of the CRISPR screening is that although the identified genes may regulate the targeted phenotype when they are artificially knocked out/activated, their expressions may not change in the real tumor or directly participate in the tumorigenesis process in real patients with cancer. In our study, by integrating the screening result obtained from melanoma cells with the TCGA data across 20 cancer types, we found that the identified lncRNAs showed a significant correlation with immune signatures in multiple cancer types. For example, *IL10RB-DT* demonstrated a strong negative correlation with immune response and immune checkpoint blockade (ICB) therapy response in several cancer types including melanoma and breast cancer. On the other hand, *LINC01198* demonstrated a high positive association with immune signatures in patients with breast cancer. Further gain- and loss-of-function investigations in breast cancer models demonstrated that *LINC01198* regulated immune response in breast cancer by interacting and activating p65. How *LINC01198* interacts with p65 and activates its phosphorylation will be our next-level study.

Together, we have shown that the CRISPR-SAM gain-of-function screening can identify lncRNA regulators as potential biomarkers for tumor immune response and immunotherapy efficacy. Functionally, we have mechanistically validated top lncRNAs’ regulation of tumor immune response. Our study represents a systematic effort to characterize lncRNAs’ role in tumor immune response.

## MATERIALS AND METHODS

### Cell lines and reagents

Human breast cancer cell line MCF-7 was purchased from the American Type Culture Collection. Human embryonic kidney (HEK) 293FT cells were purchased from Invitrogen (R70007). Peripheral blood mononuclear cells (PBMCs) transduced with GP100 TCR, MEL-526, and MEL-624 cells were provided by U. Kammula. PBMCs were cultured in RPMI 1640 (Gibco, 11875-085) supplemented with 10% heat-inactivated human AB serum (Gemini Bio-Products, 100-512), penicillin-streptomycin (100 U/ml; Lonza, 17-602E), gentamicin (50 μg/ml; Gibco, 15750-060), and 10 mM Hepes (Lonza, 17-737E). MCF-7 cells were cultured in Dulbecco’s modified Eagle’s medium (DMEM); HyClone, SH30243.01) supplemented with 10% fetal bovine serum (FBS; Corning, 35-011-CV) and 1% penicillin-streptomycin (Corning, 30-002-Cl). MEL-526 and MEL-624 cells were cultured in RPMI 1640 (HyClone, SH30027.01) supplemented with 10% FBS and 1% penicillin-streptomycin. HEK293FT cells were cultured in DMEM supplemented with GlutaMAX (Gibco, 10569010), 10% FBS, and 1% penicillin-streptomycin. The cells were treated for 24 hours with human IFN-γ (1, 5, and 25 ng/ml; Sigma-Aldrich, I17001) when necessary.

### CRISPR library preparation, lentivirus production and transfection, and vector cloning

The preparation of the library and cells followed the previously published protocol (*53*). Human CRISPR lncRNA Activation Pooled Library (SAM-3 plasmid system) was purchased from Addgene (1000000106). On the basis of ENCODE V19, the library contained 96,458 sgRNAs targeting 9744 TSSs of lncRNAs and also contained 500 control sgRNAs. Pooled sgRNA library was first amplified by electroporation. The antibiotic killing curve using zeocin, blasticidin, and hygromycin was performed before lentivirus production for all three plasmids (sgRNA library, dCas9-VP64, and MS2-P65-HSF1, respectively). The concentration for selection was indicated below: zeocin (400 μg/ml; InvivoGen, ant-zn-05), blasticidin (10 μg/ml; Sigma-Aldrich, 15205), and hygromycin (250 μg/ml; RPI, 31282-04-9). Before transducing the lncRNA library, we established the dCas9-VP64 and MS2-p65-HSF1 stably expressed MEL-526 cells, and the multiplicity of infection (MOI) of lentivirus is 0.5 and selected with antibiotics. HEK293FT cells were cultured in T225 flasks and maintained at 70% confluency. Cells for transfection were seeded at 80 to 90% confluency; transfection used Lipofectamine 2000 (Invitrogen, 11668-019) and Plus reagent (Invitrogen, 11514-015). For each T225 flask, 2250 μl of Opti-MEM (Gibco, 11058-021) was mixed with 15.3 μg of pMD2.G (Addgene, 12259), 23.4 μg of psPAX2 (Addgene, 12260), and 30.6 μg of sgRNA library plasmids. After 8 hours of transfection, the medium was changed to complete DMEM. Lentivirus was harvested after 48 and 72 hours of transfection and filtered with 0.45-μm filters. The MOI value was determined and calculated for library lentivirus by infecting the MEL-526 cells with the titer of library lentivirus and selecting by the proper antibiotic based on the killing curve. Then, MEL-526 cells transduced with CRISPR-SAM system were infected with lncRNA sgRNA library lentivirus at an MOI of 0.3, maintaining a coverage of >500 cells expressing each sgRNA. Cells were selected with zeocin for 6 days, and then, the library cells were amplified for screening.

As for the single sgRNA activation stable cell line, sgRNAs of selected lncRNAs and control NT sgRNAs were cloned into Lenti-sgRNA (MS2)-Puro plasmid (Addgene, 73795) at Bsm BI sites. The sgRNA sequence for activated target lncRNAs and control sgRNAs were listed below: sg*IL10RB-DT*-1, 5′-AGCCTTGGGAGCTGGCTGGG-3′; sg*IL10RB-DT*-2, 5′-CCCAGCGTCCGTCCATGGCG-3′; sg*LINC01198*-1, 5′-ATGAAAGACTTGCTGTTCTT-3′; sg*LINC01198*-2, 5′-TCTCTCTCTAACGCATACAA-3′; sgNT-1, 5′-CTGAAAAAGGAAGGAGTTGA-3′; and sgNT-2, 5′-AAGATGAAAGGAAAGGCGTT-3′. To generate stable MEL-526-VP64-MPH and MCF-7-VP64-MPH cells, MEL-526 and MCF-7 cells were infected with lenti dCas9-VP64-Blast (Addgene, 61425) and lenti MS2-P65-HSF1-Hygro (Addgene, 89308) lentivirus and selected with blasticidin and hygromycin. To produce sgRNA lentiviral particles, HEK293T cells were seeded into the 6-cm dish in DMEM supplemented with 10% FBS. HEK293T cells reached 80% confluency on the second day, and transfection was performed using Lipofectamine 2000 transfection reagent (Invitrogen, 11668-019) according to the manufacturer’s recommendations. gRNA plasmids (3 μg), psPAX2 (2.25 μg; Addgene, 12260), and pVSV-G (0.75 μg; Addgene, 8454) were used for each dish. The lentivirus was collected after 48 hours of transfection and filtered with 0.45-μm filters. MEL-526-VP64-MPH cells were infected with sgRNA lentivirus for 24 hours and selected with puromycin to generate stable cells.

### Screening and sgRNA sequence

The screening of candidate lncRNAs was performed using the coculture of human melanoma MEL-526 library cells and human CD8^+^ T cells. The ratio of effective T cell and tumor cell (*E*:*T*) and duration were first determined by preexperiment; the final ratio of *E*:*T* used for CRISPR screening was 0.5, and the coculture duration was 4 hours. Two biological replicates were performed (S1 and S2), and each batch used a total of 5 × 10^7^ MEL-526 library cells in a round-bottom 96-well plate. For each well, 10^5^ tumor cells and 5 × 10^4^ CD8^+^ T cells were seeded and maintained for 4 hours. The control group was the MEL-526 library cells cultured under the same condition without coculture of the T cells. After coculture, T cells were removed, and the survived tumor cells were transferred to a new culture dish. The survived tumor cells were cultured for another 7 days until the cell number reached >5 × 10^7^ cells for each screened batch.

### Genomic DNA extraction and sample preparation for NGS

The preparation of samples and primers followed the previously published protocol (*53*). Genomic DNA extraction and PCR steps were performed according to the protocol described above (*53*). Genomic DNA was harvested from the surviving cells after the screening and maintained coverage of >500 cells expressing each sgRNA using the Zymo Research Quick-DNA Midiprep Plus Kit (Zymo Research, FD4075) according to the manufacturer’s protocol. Then, the extracted genomic DNA was used for PCR using NEBNext High Fidelity 2× PCR Master Mix (NEB, M0541S). The Next-Generation sequencing (NGS) primers that amplify the sgRNA library were listed in the protocol. All five samples used 10 different forward primers, and each sample used one unique reverse primer with a specific barcode. CRISPR library plasmid used 20 ng as a template, and control and screened samples used 1 μg of genomic DNA as a template. PCR reactions were performed using the following conditions: 98°C for 3 min, 23 cycles (98°C for 10 s, 63°C for 10 s, and 72°C for 25 s), and 72°C for 2 min. We pooled the PCR products and then purified them using the QIAquick PCR Purification Kit (QIAGEN, 28106) and measured the concentration using Nanodrop. We used 2 μg of PCR purification pooled product and loaded them into 2% of agarose gel. The target size was around 280 base pairs. Then, we performed gel extraction using the QIAquick Gel Extraction Kit (QIAGEN, 28706) according to the manufacturer’s directions. Then, the PCR samples are ready for sequencing.

### Quantitation of sgRNAs and CRISPR screening analysis

sgRNA read counts were first quantified by count_spacers.py (*53*) for all five samples including the original library (P), two controls (C1 and C2) with different cultured periods, and two biological replicates of MEL-526 cells (S1 and S2) cocultured with human gp100 TCR-transduced CD8^+^ T cell for 4 hours. Then, a pseudocount of 1 was added to each sgRNA’s read count, and normalization was performed using the total read counts in the sample. The fold change for each sgRNA was obtained by dividing the normalized read count in the CD8^+^ T cell cocultured sample by the control followed by the base 2 logarithm transformation. Pearson correlation coefficients were calculated for sgRNA read counts in two replicates (S1 and S2) and sgRNA log_2_ fold changes in two comparisons (S1 versus C2 and S2 versus C2).

Using the GENCODE v19 annotation (*54*), we reannotated the Human CRISPR lncRNA Activation Pooled Library (*32*) originally built on the hg19.refGene.gtf (*54*) and CabiliSuppDataSet1_lincRNAs_transcripts.gtf (*49*). Each sgRNA sequence was considered as a DNA motif, and HOMER “find” function (*55*) was used to identify the loci of the sgRNAs. The transcripts with at least two sgRNAs mapped to their promoters (−1000 to +100 b of TSS) were considered as targets of the sgRNAs in the reannotated library.

RIGER (*56*) was used to identify candidate genes with recommended parameters described in the protocol (*53*). The frequency of sgRNAs targeting significant candidates was compared in cocultured samples and control samples using a two-tailed paired Student’s *t* test. A weighted average of RIGER *P* values was calculated for each gene in two cocultured replicates as follows

$$\text{Weighted\textbackslash{} average}\;P=\alpha \times \frac{\text{-}\log_{10}({\;p}_{rep1})-\log_{10}({\;p}_{rep2})}{2},\;\text{w}here$$

$$\alpha =\left\{ \begin{array}{cc} 2, & \text{if}\;{\;p}_{rep1}<0.05\;and\;{\;p}_{rep2}<0.05\\ 0.5, & \text{if}\;{\;p}_{rep1}\geq 0.05\;and\;{\;p}_{rep2}\geq 0.05\\ 1, & \text{o}therwise \end{array}\right.$$

MAGeCK (v0.5.9.4) (*34*) was also used for sgRNA quantification, and the MAGeCK RRA algorithm was then used for the candidate gene selection with 500 nontargeting control sgRNAs for normalization and generation of the null distribution. Parameters were set as defaulted. Candidate genes with *P* < 0.05 in both CD8^+^ T cell cocultured replicates by RIGER and MAGeCK were selected as significantly depleted/enriched genes from our CRISPR screening.

### TCGA database and analysis

TCGA (*57*) RNA-seq and clinical data were obtained from the Genomic Data Commons portal. The annotation for the transcriptome data was performed on the human reference genome GRCh38, and the gene expression level was then quantified by fragments per kilobase of transcript per million mapped reads (FPKM) upper quartile. We also obtained the 68 immune signatures from previous studies (*23*) for 10,850 TCGA patients with cancer. Spearman’s correlation coefficient was calculated between the individual lncRNA expression and immune signature scores in each cancer type. Specifically, in patients with breast cancer, the relative immune infiltration of CD8^+^ T cells, CD4^+^ T cells, DCs, neutrophils, macrophages, and B cells was estimated using the Tumor Immune Estimation Resource (*58*). Then, Spearman’s correlation between immune infiltration level and each lncRNA expression was calculated. lncRNA expression was compared between TCGA tumor samples and normal samples from Genotype-Tissue Expression (GTEx) database (*59*) using a two-tailed unpaired Student’s *t* test.

### RNA-seq analysis

MEL-526 and MCF-7 cells were transduced with individual sgRNAs targeting *IL10RB-DT* and *LINC01198* or nontargeting control sgRNAs. MEL-526 cells were treated with siRNA-targeting selected lncRNAs, transiently transfected by RNAiMAX reagent (Invitrogen, 13778-150) for 48 hours. Cells were extracted using total RNA by TRIzol; purification of RNA used the RNA Clean & Concentrator Kit (Zymo Research, 11514-015). Purified RNAs were sent to University of PIttsburgh Medical Center (UPMC) Children’s Hospital for quality control, library construction, and sequencing. Reads were mapped to the human reference genome GRCh38 using spliced transcript alignment to a reference (STAR) (*60*). Gene expression quantification and differential expression analysis were performed using RNA-seq by expectation maximization (RSEM) (*61*) and Cuffdiff (*62*) on the activation and knockdown samples against their controls.

### *LINC01198* activation signature

A *LINC01198* activation signature was generated using the RNA-seq data of the MEL-526 nontargeting control sample and *LINC01198* activation sample by a similar method previously described in (*63*). One hundred ninety up-regulated and 190 down-regulated genes with Cuffdiff *P* < 0.05 and top log_2_-transformed fold changes were extracted. We then calculated the weight *k_i_* for each selected signature gene using $\$k_{i}=\frac{w_{i}}{\max(|w|)}\$$, where *w_i_* is the Cuffdiff test statistic for the *i*th selected gene and *k_i_* ∈ [ − 1,1]. With an RNA-seq profile for a new patient, we first obtained expression *z* scores for all the genes within the sample. Then, a *LINC01198* activation signature score *S* was calculated as the weighted sum expression of the signature genes using the equation $\$S=\sum_{i=1}^{380}(k_{i}^{*}z_{i})\$$, where *z_i_* denotes the expression level of the *i*th gene. A higher *S* score indicates a higher similarity with the expression profile of *LINC01198* activation.

### Anti–PD-1 treatment patient cohorts and analysis

RNA-seq data for patients with metastatic melanoma after anti–PD-1 treatment was obtained from GSE91061 (*41*), GSE145996 (*42*), and GSE78220 (*38*). Pretreated lncRNA expression/*LINC01198* activation signature score was compared between patients with different responses to anti–PD-1 therapy using a two-tailed unpaired Student’s *t* test in patient cohorts. In each dataset, we first created the MHC-I signature using the average expression of beta-2-microglobulin (B2M), human lymphocyte antigen-A (HLA-A), HLA-B, and HLA-C and the Cytotoxic T cell lymphocyte (CTL) signature using the average expression of CD8A, CD8B, GZMA, GZMB, and Perforin 1 (PRF1). Pearson correlation coefficient was then calculated between the *LINC01198* activation and MHC-I/CTL signatures.

### Gene set enrichment analysis

GSEA was then performed on the preranked gene lists using the cancer hallmark gene set or C2 Curated gene set from the Molecular Signatures Database(MSigDB). In the TCGA data, 19,668 PCGs were ranked by their expression Spearman correlation coefficients with *LINC01198* in the breast cancer samples. In the RNA-seq data of the MEL526 and MCF-7 cell line, we ranked 20,442 PCGs by their signed log-transformed

Cuffdiff *P* values comparing the expression in the treated sample to the control sample.

### RNA interference

The siRNA treatment to knockdown *IL10RB-DT*, *LINC01198*, and control siRNA were transfected with Lipofectamine RNAiMAX (Invitrogen, 13778-150) according to the manufacturer’s directions. Sixty picomoles of siRNA was used for each well in a six-well plate. The total RNA and protein were extracted after a 48-hour treatment of siRNA. The siRNA sequences were listed as follows: siControl, Santa Cruz Biotechnology (sc-37007); si*IL10RB-DT*-1, 5′-GUCCAGUGUCUUGGGUAAU-3′; si*IL10RB-DT*-2, 5′-AGAAGAGCUAGAGGGAGUU-3′; si*LINC01198*-1: 5′-GCAAACAACUGACAGGAUA-3′; and si*LINC01198*-2, 5′-GAACCUAUGCCGUGGAGAA-3′.

### In vitro coculture assay, flow cytometry, and ELISA

The ratio of effective CD8^+^ T cells (GP100 TCR-transduced human CD8^+^ T cells were provided by the Kammula laboratory from UPMC Hillman Cancer Center) and tumor cells (MEL-526 single sgRNA activation or control NT cells) is *E*:*T* = 0.5:1. Both tumor cells and T cells were first counted, and then, 1 × 10^5^ tumor cells and 5 × 10^4^ T cells were seeded into each well of a round-bottom 96-well plate. After different time points of coculture (6, 12, 20, and 24 hours), cells and supernatant were collected and first stained with Ghost Dye (Biosciences, 13-0870-T100). Then, cells were washed with FBS stain buffer (BD, 554656) and stained with human anti-CD3^+^ (BioLegend, 344817), anti-CD4^+^ (BioLegend, 344605), anti-CD8^+^ (BioLegend, 344711), anti-CD137 (4-1BB; BioLegend, 309809), and anti–PD-1 (BioLegend, 367412) for 40 min. Cells were filtered with FBS stain buffer into tubes and analyzed by flow cytometry (Aurora, Cytek).

The supernatant collected from the coculture assay was used for enzyme-linked immunosorbent assay (ELISA). The dilution ratio of the culture supernatant was first determined. The ELISA performance of IFN-γ (R&D Systems, DY285B-05), GzmB (R&D Systems, DY2906-05), CXCL10 (R&D Systems, DY266-05), and TNF-α (R&D Systems, DY210-05) was according to the manufacturer’s directions.

### Antibodies

The antibodies were listed above for flow cytometry at in vitro coculture assay. For immunoblotting, the following antibodies were used: mouse anti–MHC-I (Santa Cruz Biotechnology, sc-55582), rabbit anti-pSTAT1 (Cell Signaling Technology, 7649), rabbit anti-STAT1 (CST, 14994), rabbit anti-IFNGR1 (Millipore, MABF753), mouse anti–β-actin (Sigma-Aldrich, A5441), rabbit anti–RIG-I (Cell Signaling Technology, 3743), rabbit anti–MDA-5 (Cell Signaling Technology, 5321), rabbit anti-MAVS (Cell Signaling Technology, 3993), rabbit anti-pIRF3 (Cell Signaling Technology, 29047), rabbit anti-IRF3 (Cell Signaling Technology, 4302), rabbit anti-p-p65 (Cell Signaling Technology, 3033), rabbit anti-p65 (Cell Signaling Technology, 8242), rabbit anti-pSTING (Cell Signaling Technology, 72971), and rabbit anti-STING (Cell Signaling Technology, 13647). Rabbit anti-p65 (Cell Signaling Technology, 8242) was used for RIP and ChIP, and normal rabbit immunoglobulin G (IgG; Cell Signaling Technology, 2729) was served as a negative control.

### RNA extraction and qRT-PCR

Total RNA was extracted and isolated using TRIzol. cDNA was synthesized from 1 μg of RNA of each sample using a reverse transcription kit (Applied Biosystems, 4368813). qRT-PCR was performed using SYBR Green PCR 2× Master Mix (Applied Biosystems, 4367659) on a QuantStudio 6 Pro Real-Time PCR System (Applied Biosystems). The relative RNA expression was determined by normalizing to glyceraldehyde-3-phosphate dehydrogenase (GAPDH). The primer sequences were listed as follows: human *IL10RB-DT*, 5′-TGCCCTACAACACCAACCCA-3′ (forward) and 5′-ATCTTCCCGTCTAACCACTG-3′ (reverse); human *LINC01198*, 5′-GGGTATTAATTCTCACGGTATAAGTG-3′ (forward) and 5′-CACCGCGTTTCACTGCTTT-3′ (reverse); human *GAPDH*, 5′-GGTGAAGGTCGGAGTCAACG-3′ (forward) and 5′-TGGGTGGAATCATATTGGAACA-3′ (reverse); human *HLA-A*, 5′-AAAAGGAGGGAGTTACACTCAGG-3′ (forward) and 5′-GCTGTGAGGGACACATCAGAG-3′ (reverse); human *HLA-B*, 5′-CTACCCTGCGGAGATCA-3′ (forward) and 5′-ACAGCCAGGCCAGCAACA-3′ (reverse); human *HLA-C*, 5′-CACACCTCTCCTTTGTGACTTCAA-3′ (forward) and 5′-CCACCTCCTCACATTATGCTAACA-3′ (reverse); human *B2M*, 5′-ATGTCTCGCTCCGTGGCCTT-3′ (forward) and 5′-GACTTTCCATTCTCTGCTGG-3′ (reverse); human *TAP1*, 5′-GCTGTTCCTGGTCCTGGTGG-3′ (forward) and 5′-TTTCGAGTGAAGGTATCGGC-3′ (reverse); human *TAP2*, 5′-CAATAGCAGCGGAGAAGGTG-3′ (forward) and 5′-CTCGGCCCCAAAACTGCGAA-3′ (reverse); human *TAPBP*, 5′-CTCAGCCTCTCCAGCCTCTT-3′ (forward) and 5′-GAGCATCTTGTCCCAGTCTC-3′ (reverse); human *ERAP1*, 5′-CGGAGACTTTCCACGGATTT-3′ (forward) and 5′-GAAGGCAGGTTCATCAAAGC-3′ (reverse); human *ERAP2*, 5′-GGTGATGGCTTTGAAGGGT-3′ (forward) and 5′-CCTGCTCTCTCTTCGTATC-3′ (reverse); human *IFNGR1*, 5′-GTCAGAGTTAAAGCCAGGGTTG-3′ (forward) and 5′-CTTCCTGCTCGTCTCCATTTAC-3′ (reverse); human *IRF1*, 5′-CATGGCTGGGACATCAACAA-3′ (forward) and 5′-TTGTATCGGCCTGTGTGAATG -3′ (reverse); human *IRF9*, 5′-AGGACCAGGA TGCTGCCTTC-3′ (forward) and 5′-TAGGGCTCAGCAACATCCA-3′ (reverse); human *CXCL-10*, 5′-GCTGCCTTATCTTTCTGACT-3′ (forward) and 5′-GGACAAAATTGGCTTGCAGG-3′ (reverse); human *IFNAR1*, 5′-GGGAATGTGACTTTTTCATTCGAT-3′ (forward) and 5′-AGTCAACCTCATACCATGAAGAAG-3′ (reverse); human *IFNAR2*, 5′-GGTTAAGAACTGTGCAAATACCAC-3′ (forward) and 5′-TCTCAAACTCTGGTGGTTCAAAAG-3′ (reverse); human *IFNA*, 5′-ACTCATACACCAGGTCACGC-3′ (forward) and 5′-CAGTGTAAAGGTGCACATGACG-3′ (reverse); human *IFNB*,*1* 5′-CTTGGATTCCTACAAAGAAGCAGC-3′ (forward) and 5′-TCCTCCTTCTGGAACTGCTGCA-3′ (reverse); human *MAVS*, 5′-ATGGTGCTCACCAAGGTGTCTG-3′ (forward) and 5′-TCTCAGAGCTGCTGTCTAGCCA-3′ (reverse); human *DDX58*, 5′-CACCTCAGTTGCTGATGAAGGC-3′ (forward) and 5′-GTCAGAAGGAAGCACTTGCTACC-3′ (reverse); human *IFI27*, 5′-CGTCCTCCATAGCAGCCAAGAT-3′ (forward) and 5′-ACCCAATGGAGCCCAGGATGAA-3′ (reverse); human *IFI44*, 5′-GTGAGGTCTGTTTTCCAAGGGC-3′ (forward) and 5′-CGGCAGGTATTTGCCATCTTTCC-3′ (reverse); human *IFITM1*, 5′-GGCTTCATAGCATTCGCCTACTC-3′ (forward) and 5′-AGATGTTCAGGCACTTGGCGGT-3′ (reverse); human *DDX60*, 5′-CTGCATGGAGAAAGTGCTGAGG-3′ (forward) and 5′-AGCACCGCATAGAGTTCTGCC-3′ (reverse); human *FOSL1*, 5′-GGAGGAAGGAACTGACCGACTT-3′ (forward) and 5′-CTCTAGGCGCTCCTTCTGCTTC-3′ (reverse); human *LAMP3*, 5′-TGGGAGCCTATTTGACCGTCTC-3′ (forward) and 5′-GCTGACAACTGGAGGCTCTGTT-3′ (reverse); human *BST2*, 5′-TCTCCTGCAACAAGAGCTGACC-3′ (forward) and 5′-TCTCTGCATCCAGGGAAGCCAT-3′ (reverse); human *SERPINB2*, 5′-GCTGTTTGGTGAGAAGTCTGCG-3′ (forward) and 5′-CTGCACATTCTAGGAAGTCTACTG-3′ (reverse); human *TIPARP*, 5′-GATTCTCAGGAGCACTTGGAAAG-3′ (forward) and 5′-TGGTGTGGACAGCCTTCGTAGT-3′ (reverse); human *IRS2*, 5′-CCTGCCCCCTGCCAACACCT-3′ (forward) and 5′-TGTGACATCCTGGTGATAAAGCC-3′ (reverse); human *NFAT5* forward, 5′-CCTAATGCCCTGATGACTCCAC-3′ (forward) and 5′-GTTTGCTGAGTTGATCCAACAGAC-3′ (reverse); human *NR4A2*, 5′-AAACTGCCCAGTGGACAAGCGT-3′ (forward) and 5′-GCTCTTCGGTTTCGAGGGCAAA-3′ (reverse); human *U6*, 5′-CTCGCTTCGGCAGCACA-3′ (forward) and 5′-AACGCTTCACGAATTTGCGT-3′ (reverse); and human *EPIC1*, 5′-TATCCCTCAGAGCTCCTGCT-3′ (forward) and 5′-AGGCTGGCAAGTGTGAATCT-3′ (reverse).

### Western blot

Total protein lysate was extracted from the cells using radioimmunoprecipitation assay lysis buffer supplemented with protease inhibitor (MCE, HY-K0010) and phosphatase inhibitor (Thermo Scientific, 1861280). Protein concentration was determined by the BCA Protein Assay Kit (Thermo Fisher Scientific, 23225). Cell lysates were prepared according to the concentration supplemented with 5× SDS loading buffer and denatured at 98°C for 10 min. Protein samples were then loaded to an SDS–polyacrylamide gel electrophoresis gel and transferred to the polyvinylidene difluoride membrane (Bio-Rad, 162-0177). Membranes were blocked in 5% nonfat milk (LabScientific, M0841) in 1× Phosphate-Buffered Saline/Tween (PBST) solution at room temperature for 1 hour. Membranes were incubated in primary antibody overnight at 4°C (antibodies listed above). On the second day, membranes were washed and incubated with 1× PBST supplemented with horseradish peroxidase–conjugated secondary antibodies for 1 hour at room temperature. The band signals were detected by chemiluminescent substrate (Thermo Fisher Scientific, F32106) and exposed to x-ray films with an AX 700LE film processor (Alphatek).

### Cell fractionation assay

MEL-526 and MEL-624 cells were isolated into cytoplasmic, nuclear, and chromatin fractions using the PARIS Kit (Thermo Fisher Scientific, AM1921). Total RNA and protein were isolated from each fraction according to the manufacturer’s directions.

### Promoter cloning and reporter assay

The 5× NF-κB–responsive elements were inserted into the pGL3-Basic Vector (Promega, E1751). NF-κB and pGL3 Basic vector (1.2 μg) were transiently transfected into MCF-7 NT or *LINC01198-*activated cells using Lipofectamine 2000; β-galactosidase (β-Gal) was used as an internal control. After 24 hours, the activities of β-Gal and luciferase were detected in a Wallac 1420 Victor2 Microplate Reader (PerkinElmer). The final activities of luciferase were normalized to indicate β-Gal activities. The 5× NF-κB–responsive elements are GGGAATTTCCGGGGACTTTCCGGGAATTTCCGGGGACTTTCCGGGAATTTCC.

### RNA immunoprecipitation

Cells were washed with 1× PBS and then treated with 0.3% formaldehyde in PBS for 10 min at room temperature. Then, 1.25 M glycine was added and swirled for 5 min at room temperature on a shaker. Cells were then washed with cold PBS three times. Lysis buffer [50 mM tris-HCl (pH 7.4), 100 mM NaCl, 1% NP-40, 0.1% SDS, and 0.5% sodium deoxycholate] supplemented with protease inhibitor (MCE, HY-K0010) and ribonuclease (RNase) inhibitor (NEB, M0314L) was added to the dish; cells were collected into tubes. Then, cells were treated with deoxyribonuclease (2 μl/ml; LifeTech, AM2239) and incubated for 20 min at room temperature. After centrifugation, the cell lysate was transferred, and concentration was determined using the BCA Protein Assay Kit (Thermo Fisher Scientific, 23225). Protein G beads (25 μl; Invitrogen, F10004D) were used and incubated with 2.5 μg of indicated primary antibodies and IgG antibody at 4°C for 2 hours. Then, the beads were washed with lysis buffer and incubated with 1 mg of protein lysate overnight at 4°C. On the second day, the beads were washed with wash buffer [20 mM tris-HCl (pH 7.4), 10 mM MgCl_2_, and 0.2% Tween 20] six times. Western blot immunoprecipitation and input samples for determining the immunoprecipitation efficiency were denatured at 98°C for 10 min supplemented with 1× SDS sample buffer. RIP and input samples were reverse–cross-linked at 70°C for 1 hour. Then, TRIzol was added to the RNA samples, and RNA extraction, reverse transcription, and qRT-PCR were performed. The IP samples were normalized to input.

### Chromatin immunoprecipitation qPCR

A total of 5 × 10^6^ cells were used for each IP sample. Protein G beads (50 μl) were first washed by block solution (0.5% BSA) and incubated with 10 μg of corresponding antibodies overnight at 4°C. Cells were first treated with 11% formaldehyde for 10 min and quenched with 1.25 M glycine for 5 min. Cells were then washed three times with cold PBS and harvested into tubes. After centrifugation, cells were lastly lysed in LB3 buffer [10 mM tris-HCl (pH 8.0), 100 mM NaCl, 1 mM EDTA, 0.5 mM EGTA, 0.1% Na-deoxycholate, and 0.5% *N*-lauroylsarcosine] supplemented with 1× protease inhibitor and sonicated to shear the chromatin to produce DNA fragment size of 0.5 to 1 kb. Beads from the previous day were washed with block solution and incubated with lysate after sonication overnight at 4°C. On the next day, beads were washed six times with wash buffer [50 mM Hepes-KOH (pH 7.6), 500 mM LiCl, 1 mM EDTA, 1% NP-40, and 0.7% Na-deoxycholate], and the protein-DNA complex was eluted and then reverse–cross-linked at 65°C water bath overnight. Then, samples were treated with RNase A (QIAGEN, 19101) and proteinase K (NEB, P8107S). DNA was extracted using phenol:chloroform:isoamyl alcohol solution (Sigma-Aldrich, P2069). qRT-qPCR was performed and analyzed. IP samples were normalized to input. The primers used were indicated as follows: human *IRF1*, 5′-TTTCCCCGAAATGACGGCAC-3′ (forward) and 5′-CCAAACACTTAGCGGGATTCC-3′ (reverse); human *STAT1*, 5′-GGAAGCCGGCGGAAATACC-3′ (forward) and 5′-GCTCAGCCAATTAGACGCGG-3′ (reverse); human *IRF3* forward, 5′-TAGTTCAACTTTCCCGCGCC-3′ (forward) and 5′-AATTGAGCGTGCACCCAG-3′ (reverse); human *IFNAR1*, 5′-CAATGGGAGCTTGGAGAAGG-3′ (forward) and 5′-GCCGCCTCTTCTGACACA-3′ (reverse); human *CXCL10*, 5′-GGGAAATTCCGTAACTTGGAGGC-3′ (forward) and 5′-GACTTAGCAAAACCTGCTGGC-3′ (reverse); human *ISG15*, 5′-GGGGAATTTCCTTCCCGACT-3′ (forward) and 5′-TCTCGGGGCTCACTCTCC-3′ (reverse); human *ISG20* forward, 5′-GCGGAGGGTGAGAAAGGAAAC-3′ (forward) and 5′-CCCTCCCCCTCCTCAGAT-3′ (reverse); human *STAT3*, 5′-TCGGCTCTTCCCTCGCTG-3′ (forward) and 5′-CGGAGCCAAGAGGAGACTGA-3′ (reverse); human *TNFAIP3* forward, 5′-GGAAAGTCCCGTGGAAATCC-3′ (forward) and 5′-GCCACGAAGACTGCAGAC-3′ (reverse); human *TRIM25*, 5′-GGCTCCCGGGAGTACCTC-3′ (forward) and 5′-CCTGAAGCCGTCAGGAAGTC-3′ (reverse); human *PPP1R15A*, 5′-GTGGTCACGCTCGGAAACTC-3′ (forward) and 5′-GCAACCCGAAGGGTGGGA-3′ (reverse); human *IRF7*, 5′-TTCCGCTGGTCGCATCCAA-3′ (forward) and 5′-GCGCTTTTATGGTGGCCAGG-3′ (reverse); human *IRF9*, 5′-AGATGCTGCTGCCCTCTAGT-3′ (forward) and 5′-GTCCCCACCCTAAGTTTCAGT-3′ (reverse); and human *CXCL1*, 5′-TCCTTCCGGACTCGGGATC-3′ (forward) and 5′-GAGATCCGCGAACCCCTT-3′ (reverse).

### Statistical analysis

The association between gene expression and overall survival rates was assessed by log-rank test (*64*) and Kaplan-Meier estimate (*65*) analyses in TCGA metastatic melanoma patient samples and E-MTAB-365 (*66*) breast cancer dataset. Analysis was performed using Python 3.8.0 and GraphPad Prism. The significance threshold was defined as *P* < 0.05. The Benjamini-Hochberg adjustment field (*67*) was used to calculate the false discovery rates.

## Notes

View/request a protocol for this paper from *Bio-protocol*.

## References

[1] J. B. Swann, M. J. Smyth,Immune surveillance of tumors. J. Clin. Invest. 117,1137–1146 (2007). 17476343 10.1172/JCI31405 PMC1857231

[2] D. Hanahan, R. A. Weinberg,Hallmarks of cancer: The next generation. Cell 144,646–674 (2011). 21376230 10.1016/j.cell.2011.02.013

[3] N. Zhang, M. J. Bevan,CD8^+^ T cells: Foot soldiers of the immune system. Immunity 35,161–168 (2011). 21867926 10.1016/j.immuni.2011.07.010 PMC3303224

[4] A. M. van der Leun, D. S. Thommen, T. N. Schumacher,CD8^+^ T cell states in human cancer: Insights from single-cell analysis. Nat. Rev. Cancer 20,218–232 (2020). 32024970 10.1038/s41568-019-0235-4 PMC7115982

[5] B. Farhood, M. Najafi, K. Mortezaee,CD8^+^ cytotoxic T lymphocytes in cancer immunotherapy: A review. J. Cell. Physiol. 234,8509–8521 (2019). 30520029 10.1002/jcp.27782

[6] N. Schumacher Ton, D. Schreiber Robert,Neoantigens in cancer immunotherapy. Science 348,69–74 (2015). 25838375 10.1126/science.aaa4971

[7] J. Koury, M. Lucero, C. Cato, L. Chang, J. Geiger, D. Henry, J. Hernandez, F. Hung, P. Kaur, G. Teskey, A. Tran,Immunotherapies: Exploiting the immune system for cancer treatment. J. Immunol. Res. 2018,9585614 (2018). 29725606 10.1155/2018/9585614 PMC5872614

[8] D. M. Pardoll,The blockade of immune checkpoints in cancer immunotherapy. Nat. Rev. Cancer 12,252–264 (2012). 22437870 10.1038/nrc3239 PMC4856023

[9] J. R. Brahmer, C. G. Drake, I. Wollner, J. D. Powderly, J. Picus, W. H. Sharfman, E. Stankevich, A. Pons, T. M. Salay, T. L. McMiller, M. M. Gilson, C. Wang, M. Selby, J. M. Taube, R. Anders, L. Chen, A. J. Korman, D. M. Pardoll, I. Lowy, S. L. Topalian,Phase I study of single-agent anti–programmed death-1 (MDX-1106) in refractory solid tumors: Safety, clinical activity, pharmacodynamics, and immunologic correlates. J. Clin. Oncol. 28,3167–3175 (2010). 20516446 10.1200/JCO.2009.26.7609 PMC4834717

[10] P. Kvistborg, D. Philips, S. Kelderman, L. Hageman, C. Ottensmeier, D. Joseph-Pietras, J. P. Welters Marij, S. van der Burg, E. Kapiteijn, O. Michielin, E. Romano, C. Linnemann, D. Speiser, C. Blank, B. Haanen John, N. Schumacher Ton,Anti–CTLA-4 therapy broadens the melanoma-reactive CD8^+^ T cell response. Sci. Transl. Med. 6,254ra128 (2014). 10.1126/scitranslmed.3008918 25232180

[11] J. Larkin, V. Chiarion-Sileni, R. Gonzalez, J. J. Grob, C. L. Cowey, C. D. Lao, D. Schadendorf, R. Dummer, M. Smylie, P. Rutkowski, P. F. Ferrucci, A. Hill, J. Wagstaff, M. S. Carlino, J. B. Haanen, M. Maio, I. Marquez-Rodas, G. A. McArthur, P. A. Ascierto, G. V. Long, M. K. Callahan, M. A. Postow, K. Grossmann, M. Sznol, B. Dreno, L. Bastholt, A. Yang, L. M. Rollin, C. Horak, F. S. Hodi, J. D. Wolchok,Combined nivolumab and ipilimumab or monotherapy in untreated melanoma. N. Engl. J. Med. 373,23–34 (2015). 26027431 10.1056/NEJMoa1504030 PMC5698905

[12] A. Rotte,Combination of CTLA-4 and PD-1 blockers for treatment of cancer. J. Exp. Clin. Cancer Res. 38,255 (2019). 31196207 10.1186/s13046-019-1259-z PMC6567914

[13] S. C. Wei, N.-A. A. S. Anang, R. Sharma, M. C. Andrews, A. Reuben, J. H. Levine, A. P. Cogdill, J. J. Mancuso, J. A. Wargo, D. Pe’er, J. P. Allison,Combination anti–CTLA-4 plus anti–PD-1 checkpoint blockade utilizes cellular mechanisms partially distinct from monotherapies. Proc. Natl. Acad. Sci. U.S.A. 116,22699–22709 (2019). 31636208 10.1073/pnas.1821218116 PMC6842624

[14] S. Kelderman, T. N. M. Schumacher, J. B. A. G. Haanen,Acquired and intrinsic resistance in cancer immunotherapy. Mol. Oncol. 8,1132–1139 (2014). 25106088 10.1016/j.molonc.2014.07.011 PMC5528612

[15] R. Bajwa, A. Cheema, T. Khan, A. Amirpour, A. Paul, S. Chaughtai, S. Patel, T. Patel, J. Bramson, V. Gupta, M. Levitt, A. Asif, M. A. Hossain,Adverse effects of immune checkpoint inhibitors (programmed death-1 inhibitors and cytotoxic T-lymphocyte-associated protein-4 inhibitors): Results of a retrospective study. J. Clin. Med. Res. 11,225–236 (2019). 30937112 10.14740/jocmr3750 PMC6436564

[16] A. M. Schmitt, H. Y. Chang,Long noncoding RNAs in cancer pathways. Cancer Cell 29,452–463 (2016). 27070700 10.1016/j.ccell.2016.03.010 PMC4831138

[17] P. Wu, X. Zuo, H. Deng, X. Liu, L. Liu, A. Ji,Roles of long noncoding RNAs in brain development, functional diversification and neurodegenerative diseases. Brain Res. Bull. 97,69–80 (2013). 23756188 10.1016/j.brainresbull.2013.06.001

[18] M. Kitagawa, K. Kitagawa, Y. Kotake, H. Niida, T. Ohhata,Cell cycle regulation by long non-coding RNAs. Cell. Mol. Life Sci. 70,4785–4794 (2013). 23880895 10.1007/s00018-013-1423-0 PMC3830198

[19] M. Jiang, S. Zhang, Z. Yang, H. Lin, J. Zhu, L. Liu, W. Wang, S. Liu, W. Liu, Y. Ma, L. Zhang, X. Cao,Self-recognition of an inducible host lncRNA by RIG-I feedback restricts innate immune response. Cell 173,906–919.e13 (2018). 29706547 10.1016/j.cell.2018.03.064

[20] Q. Xie, S. Chen, R. Tian, X. Huang, R. Deng, B. Xue, Y. Qin, Y. Xu, J. Wang, M. Guo, J. Chen, S. Tang, G. Li, H. Zhu,Long noncoding RNA ITPRIP-1 positively regulates the innate immune response through promotion of oligomerization and activation of MDA5. J. Virol. 92,e00507-18 (2018). 29899107 10.1128/JVI.00507-18 PMC6096792

[21] P. Wang, Y. Xue, Y. Han, L. Lin, C. Wu, S. Xu, Z. Jiang, J. Xu, Q. Liu, X. Cao,The STAT3-binding long noncoding RNA lnc-DC controls human dendritic cell differentiation. Science 344,310–313 (2014). 24744378 10.1126/science.1251456

[22] D. Huang, J. Chen, L. Yang, Q. Ouyang, J. Li, L. Lao, J. Zhao, J. Liu, Y. Lu, Y. Xing, F. Chen, F. Su, H. Yao, Q. Liu, S. Su, E. Song,NKILA lncRNA promotes tumor immune evasion by sensitizing T cells to activation-induced cell death. Nat. Immunol. 19,1112–1125 (2018). 30224822 10.1038/s41590-018-0207-y

[23] W. Guo, Y. Wang, M. Yang, Z. Wang, Y. Wang, S. Chaurasia, Z. Wu, M. Zhang, S. Yadav Ghanshyam, S. Rathod, F. Concha-Benavente, C. Fernandez, S. Li, W. Xie, L. Ferris Robert, S. Kammula Udai, B. Lu, D. Yang,LincRNA-immunity landscape analysis identifies EPIC1 as a regulator of tumor immune evasion and immunotherapy resistance. Sci. Adv. 7,eabb3555 (2021). 33568470 10.1126/sciadv.abb3555 PMC7875530

[24] E. H. Yau, I. R. Kummetha, G. Lichinchi, R. Tang, Y. Zhang, T. M. Rana,Genome-wide CRISPR screen for essential cell growth mediators in mutant KRAS colorectal cancers. Cancer Res. 77,6330–6339 (2017). 28954733 10.1158/0008-5472.CAN-17-2043 PMC5690866

[25] S. Chen, N. E. Sanjana, K. Zheng, O. Shalem, K. Lee, X. Shi, D. A. Scott, J. Song, J. Q. Pan, R. Weissleder, H. Lee, F. Zhang, P. A. Sharp,Genome-wide CRISPR screen in a mouse model of tumor growth and metastasis. Cell 160,1246–1260 (2015). 25748654 10.1016/j.cell.2015.02.038 PMC4380877

[26] A. C. Bester, J. D. Lee, A. Chavez, Y.-R. Lee, D. Nachmani, S. Vora, J. Victor, M. Sauvageau, E. Monteleone, J. L. Rinn, P. Provero, G. M. Church, J. G. Clohessy, P. P. Pandolfi,An integrated genome-wide CRISPRa approach to functionalize lncRNAs in drug resistance. Cell 173,649–664.e20 (2018). 29677511 10.1016/j.cell.2018.03.052 PMC6061940

[27] S. Xu, M. Zhan, C. Jiang, M. He, L. Yang, H. Shen, S. Huang, X. Huang, R. Lin, Y. Shi, Q. Liu, W. Chen, M. Mohan, J. Wang,Genome-wide CRISPR screen identifies ELP5 as a determinant of gemcitabine sensitivity in gallbladder cancer. Nat. Commun. 10,5492 (2019). 31792210 10.1038/s41467-019-13420-x PMC6889377

[28] S. J. Liu, A. H. Max, W. C. Seung, S. B. Harjus, M. Malatesta, D. He, J. A. Frank, E. V. Jacqueline, Y. C. Min, Y. Chen, A. M. Mohammad, P. O. Michael, A. G. Luke, R. C. Bruce, Y. C. Howard, S. W. Jonathan, A. L. Daniel,CRISPRi-based genome-scale identification of functional long noncoding RNA loci in human cells. Science 355,eaah7111 (2017). 10.1126/science.aah7111 PMC5394926 27980086

[29] Y. Liu, Z. Cao, Y. Wang, Y. Guo, P. Xu, P. Yuan, Z. Liu, Y. He, W. Wei,Genome-wide screening for functional long noncoding RNAs in human cells by Cas9 targeting of splice sites. Nat. Biotechnol. 36,1203–1210 (2018). 10.1038/nbt.4283 30395134

[30] R. Esposito, N. Bosch, A. Lanzós, T. Polidori, C. Pulido-Quetglas, R. Johnson,Hacking the cancer genome: Profiling therapeutically actionable long non-coding RNAs using CRISPR-Cas9 screening. Cancer Cell 35,545–557 (2019). 30827888 10.1016/j.ccell.2019.01.019

[31] J. G. Doench,Am I ready for CRISPR? A user’s guide to genetic screens. Nat. Rev. Genet. 19,67–80 (2018). 29199283 10.1038/nrg.2017.97

[32] J. Joung, J. M. Engreitz, S. Konermann, O. O. Abudayyeh, V. K. Verdine, F. Aguet, J. S. Gootenberg, N. E. Sanjana, J. B. Wright, C. P. Fulco, Y.-Y. Tseng, C. H. Yoon, J. S. Boehm, E. S. Lander, F. Zhang,Genome-scale activation screen identifies a lncRNA locus regulating a gene neighbourhood. Nature 548,343–346 (2017). 28792927 10.1038/nature23451 PMC5706657

[33] M.-B. Nielsen, V. Monsurro, S. A. Migueles, E. Wang, A. Perez-Diez, K.-H. Lee, U. Kammula, S. A. Rosenberg, F. M. Marincola,Status of activation of circulating vaccine-elicited CD8^+^ T cells. J. Immunol. 165,2287–2296 (2000). 10925318 10.4049/jimmunol.165.4.2287

[34] W. Li, H. Xu, T. Xiao, L. Cong, M. I. Love, F. Zhang, R. A. Irizarry, J. S. Liu, M. Brown, X. S. Liu,MAGeCK enables robust identification of essential genes from genome-scale CRISPR/Cas9 knockout screens. Genome Biol. 15,554 (2014). 25476604 10.1186/s13059-014-0554-4 PMC4290824

[35] D. Dersh, J. D. Phelan, M. E. Gumina, B. Wang, J. H. Arbuckle, J. Holly, R. J. Kishton, T. E. Markowitz, M. O. Seedhom, N. Fridlyand, G. W. Wright, D. W. Huang, M. Ceribelli, C. J. Thomas, J. B. Lack, N. P. Restifo, T. M. Kristie, L. M. Staudt, J. W. Yewdell,Genome-wide screens identify lineage- and tumor-specific genes modulating MHC-I- and MHC-II-restricted immunosurveillance of human lymphomas. Immunity 54,116–131.e10 (2021). 33271120 10.1016/j.immuni.2020.11.002 PMC7874576

[36] C. Gstalder, D. Liu, D. Miao, B. Lutterbach, A. L. DeVine, C. Lin, M. Shettigar, P. Pancholi, E. I. Buchbinder, S. L. Carter, M. P. Manos, V. Rojas-Rudilla, R. Brennick, E. Gjini, P.-H. Chen, A. Lako, S. Rodig, C. H. Yoon, G. J. Freeman, D. A. Barbie, F. S. Hodi, W. Miles, E. M. Van Allen, R. Haq,Inactivation of *Fbxw7* impairs dsRNA sensing and confers resistance to PD-1 blockade. Cancer Discov. 10,1296–1311 (2020). 32371478 10.1158/2159-8290.CD-19-1416 PMC8802534

[37] J. Deng, A. Thennavan, I. Dolgalev, T. Chen, J. Li, A. Marzio, J. T. Poirier, D. H. Peng, M. Bulatovic, S. Mukhopadhyay, H. Silver, E. Papadopoulos, V. Pyon, C. Thakurdin, H. Han, F. Li, S. Li, H. Ding, H. Hu, Y. Pan, V. Weerasekara, B. Jiang, E. S. Wang, I. Ahearn, M. Philips, T. Papagiannakopoulos, A. Tsirigos, E. Rothenberg, J. Gainor, G. J. Freeman, C. M. Rudin, N. S. Gray, P. S. Hammerman, M. Pagano, J. V. Heymach, C. M. Perou, N. Bardeesy, K.-K. Wong,ULK1 inhibition overcomes compromised antigen presentation and restores antitumor immunity in LKB1-mutant lung cancer. Nat. Cancer 2,503–514 (2021). 34142094 10.1038/s43018-021-00208-6 PMC8205437

[38] W. Hugo, J. M. Zaretsky, L. Sun, C. Song, B. H. Moreno, S. Hu-Lieskovan, B. Berent-Maoz, J. Pang, B. Chmielowski, G. Cherry, E. Seja, S. Lomeli, X. Kong, M. C. Kelley, J. A. Sosman, D. B. Johnson, A. Ribas, R. S. Lo,Genomic and transcriptomic features of response to anti-PD-1 therapy in metastatic melanoma. Cell 165,35–44 (2016). 26997480 10.1016/j.cell.2016.02.065 PMC4808437

[39] S. Osuch, T. Laskus, H. Berak, K. Perlejewski, K. J. Metzner, M. Paciorek, M. Radkowski, K. Caraballo Cortés,Decrease of T-cells exhaustion markers programmed cell death-1 and T-cell immunoglobulin and mucin domain-containing protein 3 and plasma IL-10 levels after successful treatment of chronic hepatitis C. Sci. Rep. 10,16060 (2020). 32994477 10.1038/s41598-020-73137-6 PMC7524731

[40] F. Zhou,Molecular mechanisms of IFN-γ to up-regulate MHC class I antigen processing and presentation. Int. Rev. Immunol. 28,239–260 (2009). 19811323 10.1080/08830180902978120

[41] N. Riaz, J. J. Havel, V. Makarov, A. Desrichard, W. J. Urba, J. S. Sims, F. S. Hodi, S. Martín-Algarra, R. Mandal, W. H. Sharfman, S. Bhatia, W.-J. Hwu, T. F. Gajewski, C. L. Slingluff Jr., D. Chowell, S. M. Kendall, H. Chang, R. Shah, F. Kuo, L. G. T. Morris, J.-W. Sidhom, J. P. Schneck, C. E. Horak, N. Weinhold, T. A. Chan,Tumor and microenvironment evolution during immunotherapy with nivolumab. Cell 171,934–949.e16 (2017). 29033130 10.1016/j.cell.2017.09.028 PMC5685550

[42] C. M. Amato, J. D. Hintzsche, K. Wells, A. Applegate, N. T. Gorden, V. M. Vorwald, R. P. Tobin, K. Nassar, Y. G. Shellman, J. Kim, T. M. Medina, M. Rioth, K. D. Lewis, M. D. McCarter, R. Gonzalez, A.-C. Tan, W. A. Robinson,Pre-treatment mutational and transcriptomic landscape of responding metastatic melanoma patients to anti-PD1 immunotherapy. Cancers (Basel) 12,1943 (2020). 32708981 10.3390/cancers12071943 PMC7409244

[43] M. Karin, Y. Yamamoto, Q. M. Wang,The IKK NF-κB system: A treasure trove for drug development. Nat. Rev. Drug Discov. 3,17–26 (2004). 14708018 10.1038/nrd1279

[44] L. M. Pfeffer,The role of nuclear factor κB in the interferon response. J. Interferon Cytokine Res. 31,553–559 (2011). 21631354 10.1089/jir.2011.0028 PMC3128784

[45] L. C. Platanias,Mechanisms of type-I- and type-II-interferon-mediated signalling. Nat. Rev. Immunol. 5,375–386 (2005). 15864272 10.1038/nri1604

[46] M. S. Hayden, S. Ghosh,Regulation of NF-κB by TNF family cytokines. Semin. Immunol. 26,253–266 (2014). 24958609 10.1016/j.smim.2014.05.004 PMC4156877

[47] M. K. Iyer, Y. S. Niknafs, R. Malik, U. Singhal, A. Sahu, Y. Hosono, T. R. Barrette, J. R. Prensner, J. R. Evans, S. Zhao, A. Poliakov, X. Cao, S. M. Dhanasekaran, Y.-M. Wu, D. R. Robinson, D. G. Beer, F. Y. Feng, H. K. Iyer, A. M. Chinnaiyan,The landscape of long noncoding RNAs in the human transcriptome. Nat. Genet. 47,199–208 (2015). 25599403 10.1038/ng.3192 PMC4417758

[48] A. E. Kornienko, C. P. Dotter, P. M. Guenzl, H. Gisslinger, B. Gisslinger, C. Cleary, R. Kralovics, F. M. Pauler, D. P. Barlow,Long non-coding RNAs display higher natural expression variation than protein-coding genes in healthy humans. Genome Biol. 17,14 (2016). 26821746 10.1186/s13059-016-0873-8 PMC4731934

[49] M. N. Cabili, M. N. Cabili, C. Trapnell, L. Goff, M. Koziol, B. Tazon-Vega, A. Regev, J. L. Rinn,Integrative annotation of human large intergenic noncoding RNAs reveals global properties and specific subclasses. Genes Dev. 25,1915–1927 (2011). 21890647 10.1101/gad.17446611 PMC3185964

[50] J. G. Doench, N. Fusi, M. Sullender, M. Hegde, E. W. Vaimberg, K. F. Donovan, I. Smith, Z. Tothova, C. Wilen, R. Orchard, H. W. Virgin, J. Listgarten, D. E. Root,Optimized sgRNA design to maximize activity and minimize off-target effects of CRISPR-Cas9. Nat. Biotechnol. 34,184–191 (2016). 26780180 10.1038/nbt.3437 PMC4744125

[51] K. R. Sanson, R. E. Hanna, M. Hegde, K. F. Donovan, C. Strand, M. E. Sullender, E. W. Vaimberg, A. Goodale, D. E. Root, F. Piccioni, J. G. Doench,Optimized libraries for CRISPR-Cas9 genetic screens with multiple modalities. Nat. Commun. 9,5416 (2018). 30575746 10.1038/s41467-018-07901-8 PMC6303322

[52] Q. Cao, J. Ma, C.-H. Chen, H. Xu, Z. Chen, W. Li, X. S. Liu,CRISPR-FOCUS: A web server for designing focused CRISPR screening experiments. PLOS ONE 12,e0184281 (2017). 28873439 10.1371/journal.pone.0184281 PMC5584922

[53] J. Joung, S. Konermann, J. S. Gootenberg, O. O. Abudayyeh, R. J. Platt, M. D. Brigham, N. E. Sanjana, F. Zhang,Genome-scale CRISPR-Cas9 knockout and transcriptional activation screening. Nat. Protoc. 12,828–863 (2017). 28333914 10.1038/nprot.2017.016 PMC5526071

[54] P. A. Fujita, B. Rhead, A. S. Zweig, A. S. Hinrichs, D. Karolchik, M. S. Cline, M. Goldman, G. P. Barber, H. Clawson, A. Coelho, M. Diekhans, T. R. Dreszer, B. M. Giardine, R. A. Harte, J. Hillman-Jackson, F. Hsu, V. Kirkup, R. M. Kuhn, K. Learned, C. H. Li, L. R. Meyer, A. Pohl, B. J. Raney, K. R. Rosenbloom, K. E. Smith, D. Haussler, W. J. Kent,The UCSC genome browser database: Update 2011. Nucleic Acids Res. 39,D876–D882 (2011). 20959295 10.1093/nar/gkq963 PMC3242726

[55] S. Heinz, C. Benner, N. Spann, E. Bertolino, Y. C. Lin, P. Laslo, J. X. Cheng, C. Murre, H. Singh, C. K. Glass,Simple combinations of lineage-determining transcription factors prime cis-regulatory elements required for macrophage and B cell identities. Mol. Cell 38,576–589 (2010). 20513432 10.1016/j.molcel.2010.05.004 PMC2898526

[56] B. Luo, H. W. Cheung, A. Subramanian, T. Sharifnia, M. Okamoto, X. Yang, G. Hinkle, J. S. Boehm, R. Beroukhim, B. A. Weir, C. Mermel, D. A. Barbie, T. Awad, X. Zhou, T. Nguyen, B. Piqani, C. Li, T. R. Golub, M. Meyerson, N. Hacohen, W. C. Hahn, E. S. Lander, D. M. Sabatini, D. E. Root,Highly parallel identification of essential genes in cancer cells. Proc. Natl. Acad. Sci. U.S.A. 105,20380–20385 (2008). 19091943 10.1073/pnas.0810485105 PMC2629277

[57] The Cancer Genome Atlas Research Network, J. N. Weinstein, E. A. Collisson, G. B. Mills, K. R. M. Shaw, B. A. Ozenberger, K. Ellrott, I. Shmulevich, C. Sander, J. M. Stuart,The cancer genome atlas pan-cancer analysis project. Nat. Genet. 45,1113–1120 (2013). 24071849 10.1038/ng.2764 PMC3919969

[58] T. Li, J. Fu, Z. Zeng, D. Cohen, J. Li, Q. Chen, B. Li, X. S. Liu,TIMER2.0 for analysis of tumor-infiltrating immune cells. Nucleic Acids Res. 48,W509–W514 (2020). 32442275 10.1093/nar/gkaa407 PMC7319575

[59] GTEx Consortium,The genotype-tissue expression (GTEx) project. Nat. Genet. 45,580–585 (2013). 23715323 10.1038/ng.2653 PMC4010069

[60] A. Dobin, C. A. Davis, F. Schlesinger, J. Drenkow, C. Zaleski, S. Jha, P. Batut, M. Chaisson, T. R. Gingeras,STAR: Ultrafast universal RNA-seq aligner. Bioinformatics 29,15–21 (2013). 23104886 10.1093/bioinformatics/bts635 PMC3530905

[61] B. Li, C. N. Dewey,RSEM: Accurate transcript quantification from RNA-Seq data with or without a reference genome. BMC Bioinformatics 12,323 (2011). 21816040 10.1186/1471-2105-12-323 PMC3163565

[62] S. Anders, W. Huber,Differential expression analysis for sequence count data. Genome Biol. 11,R106 (2010). 20979621 10.1186/gb-2010-11-10-r106 PMC3218662

[63] S. S. Gu, W. Zhang, X. Wang, P. Jiang, N. Traugh, Z. Li, C. Meyer, B. Stewig, Y. Xie, X. Bu, M. P. Manos, A. Font-Tello, E. Gjini, A. Lako, K. Lim, J. Conway, A. K. Tewari, Z. Zeng, A. D. Sahu, C. Tokheim, J. L. Weirather, J. Fu, Y. Zhang, B. Kroger, J. H. Liang, P. Cejas, G. J. Freeman, S. Rodig, H. W. Long, B. E. Gewurz, F. S. Hodi, M. Brown, X. S. Liu,Therapeutically increasing MHC-I expression potentiates immune checkpoint blockade. Cancer Discov. 11,1524–1541 (2021). 33589424 10.1158/2159-8290.CD-20-0812 PMC8543117

[64] J. M. Bland, D. G. Altman,The logrank test. BMJ 328,1073 (2004). 15117797 10.1136/bmj.328.7447.1073 PMC403858

[65] E. L. Kaplan, P. Meier,Nonparametric estimation from incomplete observations. J. Am. Stat. Assoc. 53,457–481 (1958).

[66] T. Rème, D. Hose, C. Theillet, B. Klein,Modeling risk stratification in human cancer. Bioinformatics 29,1149–1157 (2013). 23493321 10.1093/bioinformatics/btt124

[67] Y. Benjamini, Y. Hochberg,Controlling the false discovery rate: A practical and powerful approach to multiple testing. J. R. Stat. Soc. Series B Stat. Methodol. 57,289–300 (1995).

