# Supplementary material for: Genome-wide gain-of-function screening characterized lncRNA regulators for tumor immune response
Source: Sci Adv. 2022 Dec 7;8(49):eadd0005. doi: 10.1126/sciadv.add0005 (PMC9728976; doi:10.1126/sciadv.add0005)
Supplement: Supplementary file 1 — Figs. S1 to S7 [file sciadv.add0005_sm.pdf]

Science Advances
AAAS

Supplementary Materials for

Genome-wide gain-of-function screening characterized lncRNA regulators for tumor immune response

Yifei Wang *et al.*

Corresponding author: Da Yang, dyang@pitt.edu; Min Zhang, miz45@pitt.edu

Sci. Adv. **8**, eadd0005 (2022)
DOI: 10.1126/sciadv.add0005

The PDF file includes:

Figs. S1 to S7
Legends for data S1 to S3

Other Supplementary Material for this manuscript includes the following:

Data S1 to S3

figure S1

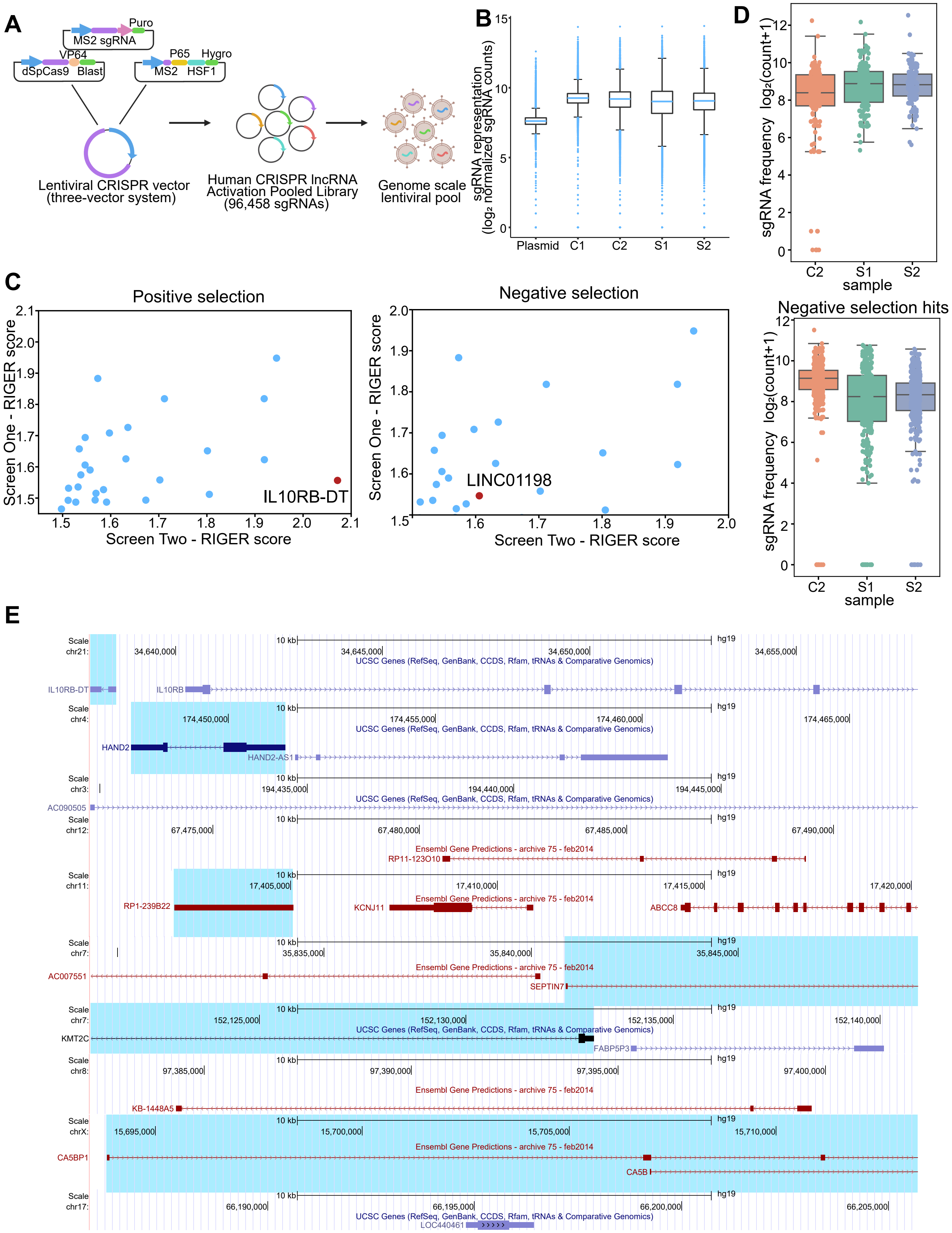
A
Puro
MS2 sgRNA
VP64
dSpCas9 Blast
P65 Hygro
MS2 HSF1
Lentiviral CRISPR vector
(three-vector system)
Human CRISPR lncRNA
Activation Pooled Library
(96,458 sgRNAs)
Genome scale
lentiviral pool
B
sgRNA representation
(log₂ normalized sgRNA counts)
Plasmid
C1
C2
S1
S2
D
Positive selection hits
sgRNA frequency log₂(count+1)
sample
Negative selection hits
C
Positive selection
Screen One - RIGER score
Screen Two - RIGER score
IL10RB-DT
Negative selection
LINC01198
E
Scale
chr21:
10 kb
hg19
UCSC Genes (RefSeq, GenBank, CCDS, Rfam, tRNAs & Comparative Genomics)
IL10RB-DT
IL10RB
chr4:
HAND2
HAND2-AS1
chr3:
AC090505
chr12:
Ensembl Gene Predictions - archive 75 - feb2014
RP11-123O10
chr11:
RP1-239B22
KCNJ11
ABCC8
chr7:
AC007551
SEPTIN7
KMT2C
FABP5P3
chr8:
KB-1448A5
chrX:
CA5BP1
CA5B
chr17:
LOC440461

F

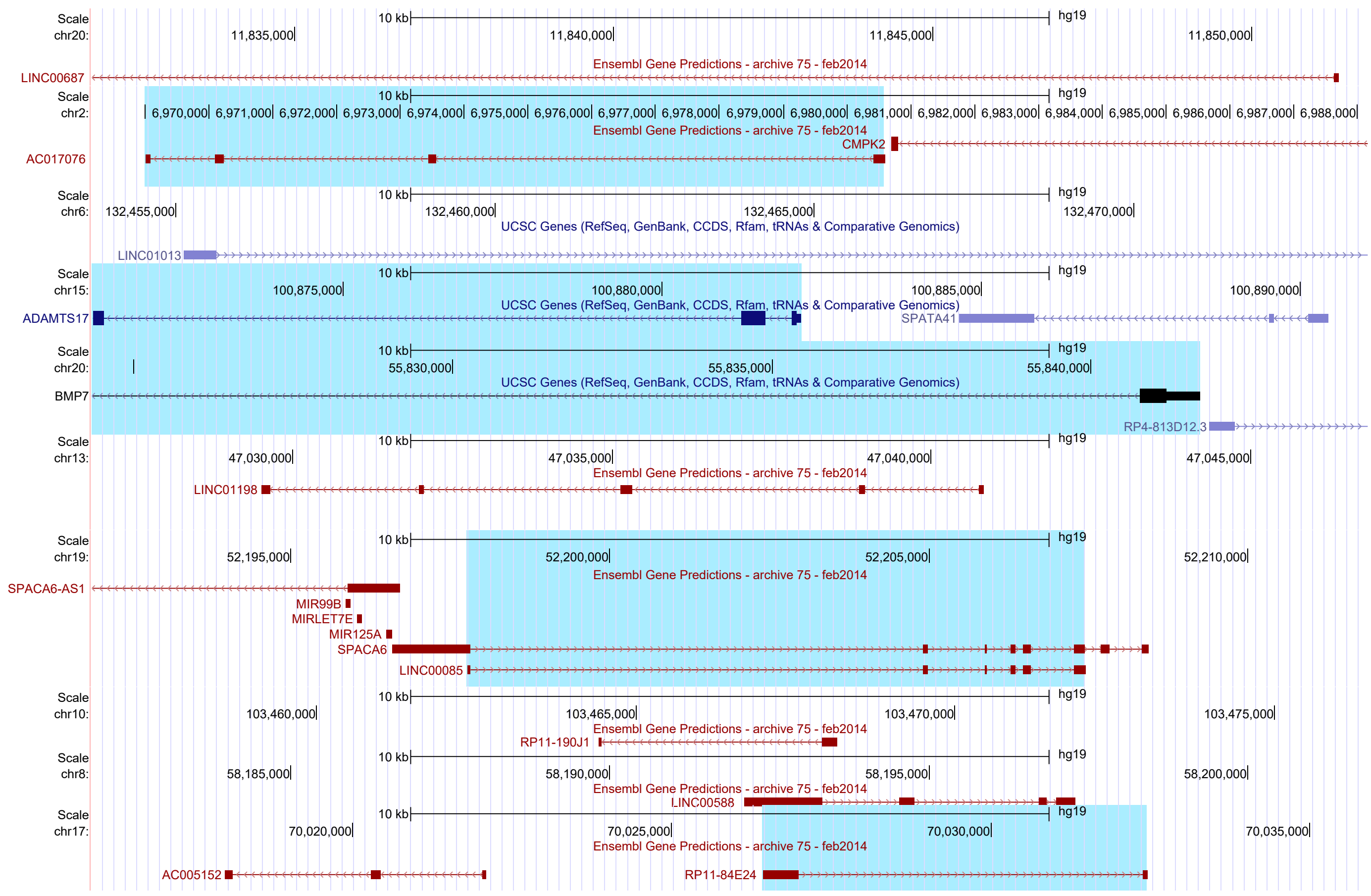

fig. S1. Genome-wide CRISPRa Screen identifies regulatory lncRNAs for tumor cell response to CD8$^+$ T cell-mediated cytotoxicity. (**A**) The three-vector system containing dCas9-VP64, MS2-P65-HSF1, and sgRNA library was transduced into MEL-526 cells to obtain genome-scale activation. (**B**) Box plots of sgRNA frequencies in the five samples. (**C**) RIGER score in the two comparisons (S1 vs. C2 and S2 vs. C2) for top-ranked genes in positive and negative selections respectively. (**D**) Frequencies of sgRNAs for significant positive and negative selections in control and co-cultured samples. (positive selection: C2 vs S1: $p<0.0001$, C2 vs S2: $p<0.0001$, negative selection: C2 vs S1: $p<0.0001$, C2 vs S2: $p<0.0001$). (**E** and **F**) The location of top 10 hit genes for positive selection (E) and negative selection (F) displayed in UCSC genome browser. (If there are other neighbor genes around 20 kb of the hit gene, the hit gene will be highlighted).

figure S2

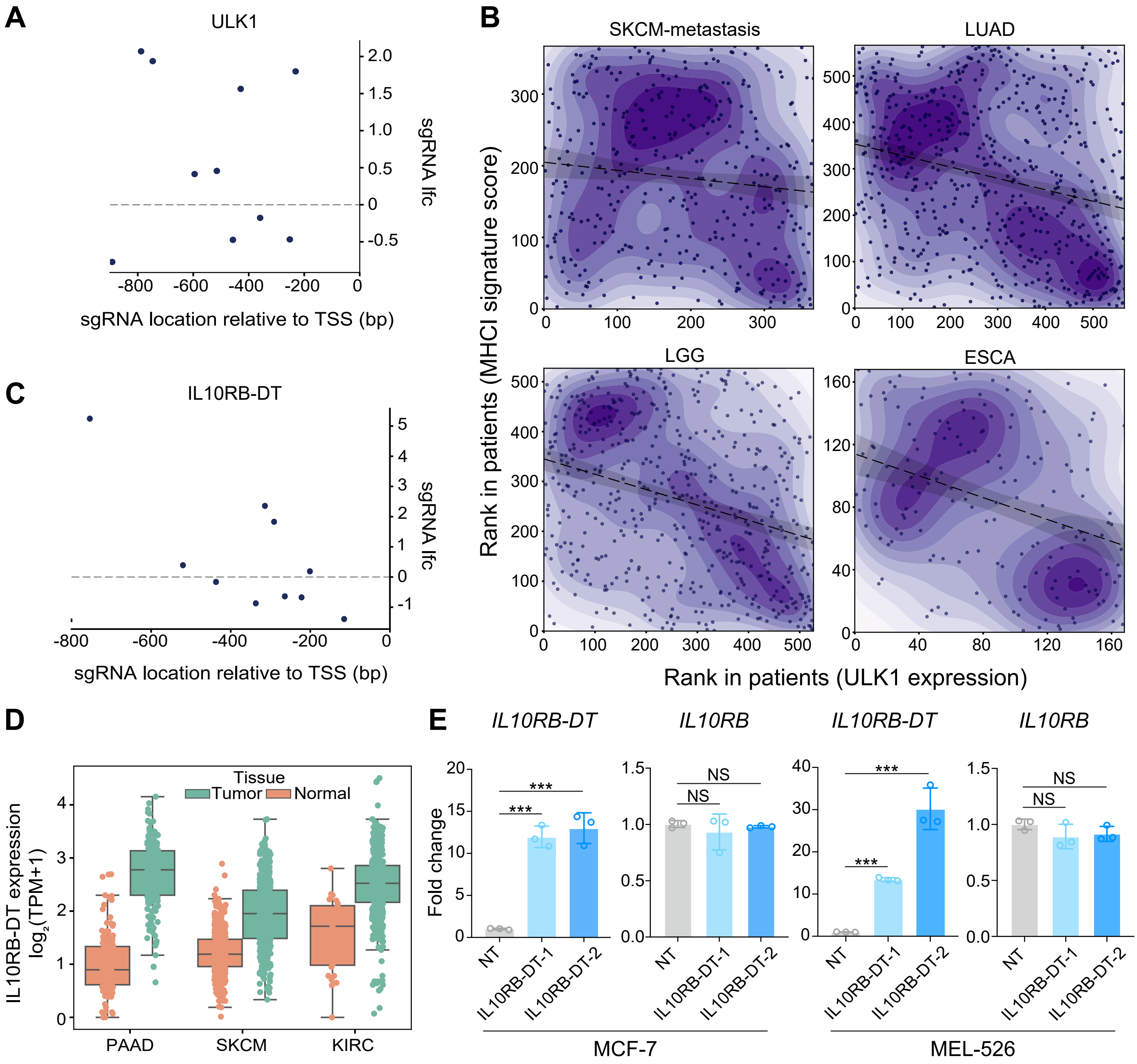

fig. S2. CRISPRa screen characterizes protein-coding gene ULK1 and lncRNA *IL10RB-DT* as tumor immune response suppressors. (**A** and **C**) The location and log_2-transformed fold change of sgRNAs targeting ULK1 and *IL10RB-DT* respectively. (**B**) ULK1's expression negatively correlated with MHC-I signature in TCGA metastatic Melanoma (SKCM), Lung Adenocarcinoma (LUAD), Brain Low Grade Glioma (LGG), and Esophageal Carcinoma (ESCA) patients. (**D**) *IL10RB-DT* is highly expressed in TCGA Pancreatic Adenocarcinoma (PAAD) ($p = 1.3E^{-85}$), SKCM ($p = 9.5E^{-104}$), and Kidney Renal Clear Cell Carcinoma (KIRC) ($p = 1.8E^{-17}$) samples compared to GTEx normal tissue samples. (**E**) Real-time PCR detection of significant *IL10RB-DT* activation in MEL-526-dCas9-VP64-MPH and MCF-7-dCas9-VP64-MPH cells transfected with sgRNAs targeting *IL10RB-DT* compared to non-targeting sgRNA control (NT); the expression of *IL10RB* has no significant changes (n = 3 independent experiment, mean ± s.d., two-tailed t-test, unpaired). Statistical significance: *p < 0.05, **p < 0.01, ***p < 0.001.

figure S3

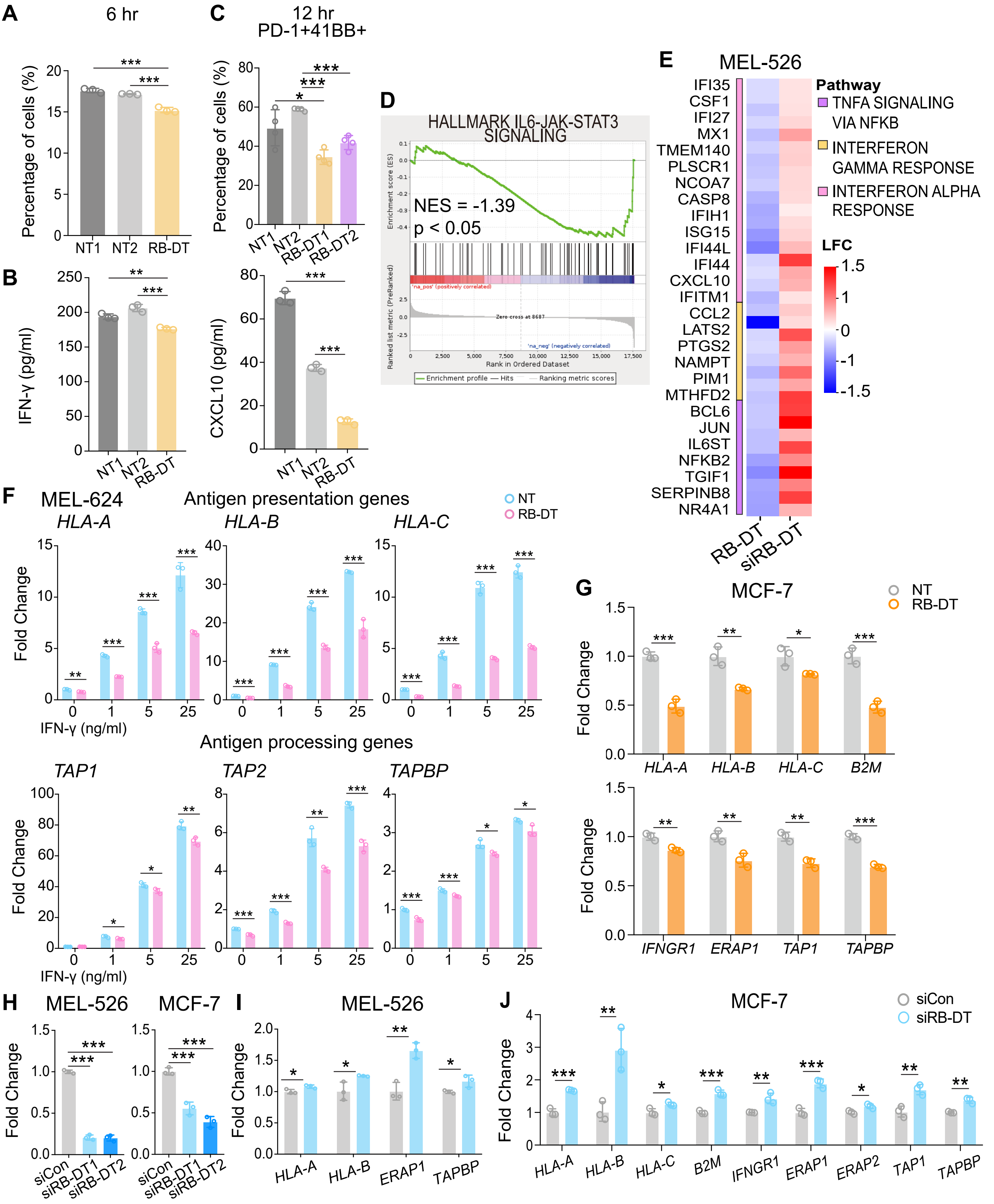

fig. S3. *IL10RB-DT* suppresses tumor antigen presentation via IFN-γ-JAK-STAT1 signaling. (**A**) Quantification of gp100 TCR-transduced CD8$^+$ T activation level after co-culture with MEL-526 cells that transfected with either non-targeting sgRNA (NT) control or single sgRNA activated *IL10RB-DT* (RB-DT) for 6 h. The data shown represent one of three independent experiments with similar results (mean ± s.d., two-tailed t-test, unpaired). (**B**) IFN-γ and CXCL10 secretion level from 6 h co-culture (A) supernatant detected by ELISA. Data shown represent one of three independent experiments with similar results (mean ± s.d., two-tailed t-test, unpaired).

(**C**) Quantification of gp100 TCR-transduced $CD8^+$ T activation level for 12 h co-culture. The data shown are representative of three independent experiments (mean ± s.d., two-tailed t-test, unpaired). (**D**) Genes in the IL6-JAK-STAT3 signaling pathway showed decreased expression in *IL10RB-DT* activated MEL526 cells compared to the control sample. (**E**) Gene expression in the three immune-related pathways is negatively regulated by *IL10RB-DT* in MEL526 cells (LFC: $\log_2$-transformed fold-change). (**F** and **G)** Real-time PCR result showing the mRNA expression level of antigen presentation and processing genes in MEL-624 control (NT) and *IL10RB-DT* activated (RB-DT) cells (F) treated with IFN-γ (0, 1, 5, 25 ng/ml) for 24 h and in MCF-7 (G) control (NT) and *IL10RB-DT* (RB-DT) activated cells (n =3 independent experiment, mean ± s.d., two-tailed t-test, unpaired). (**H**-**J**) Real-time PCR result showing the knockdown efficiency of *IL10RB-DT* by siRNA treatment in MEL-526 and MCF-7 cells (H) and antigen presentation and processing genes expression level after *IL10RB-DT* or control siRNA treatment in MEL-526 (I) and MCF-7 (J) cells (n = 3 independent experiment, mean ± s.d., two-tailed t-test, unpaired). Statistical significance: *p < 0.05, **p < 0.01, ***p < 0.001.

figure S4

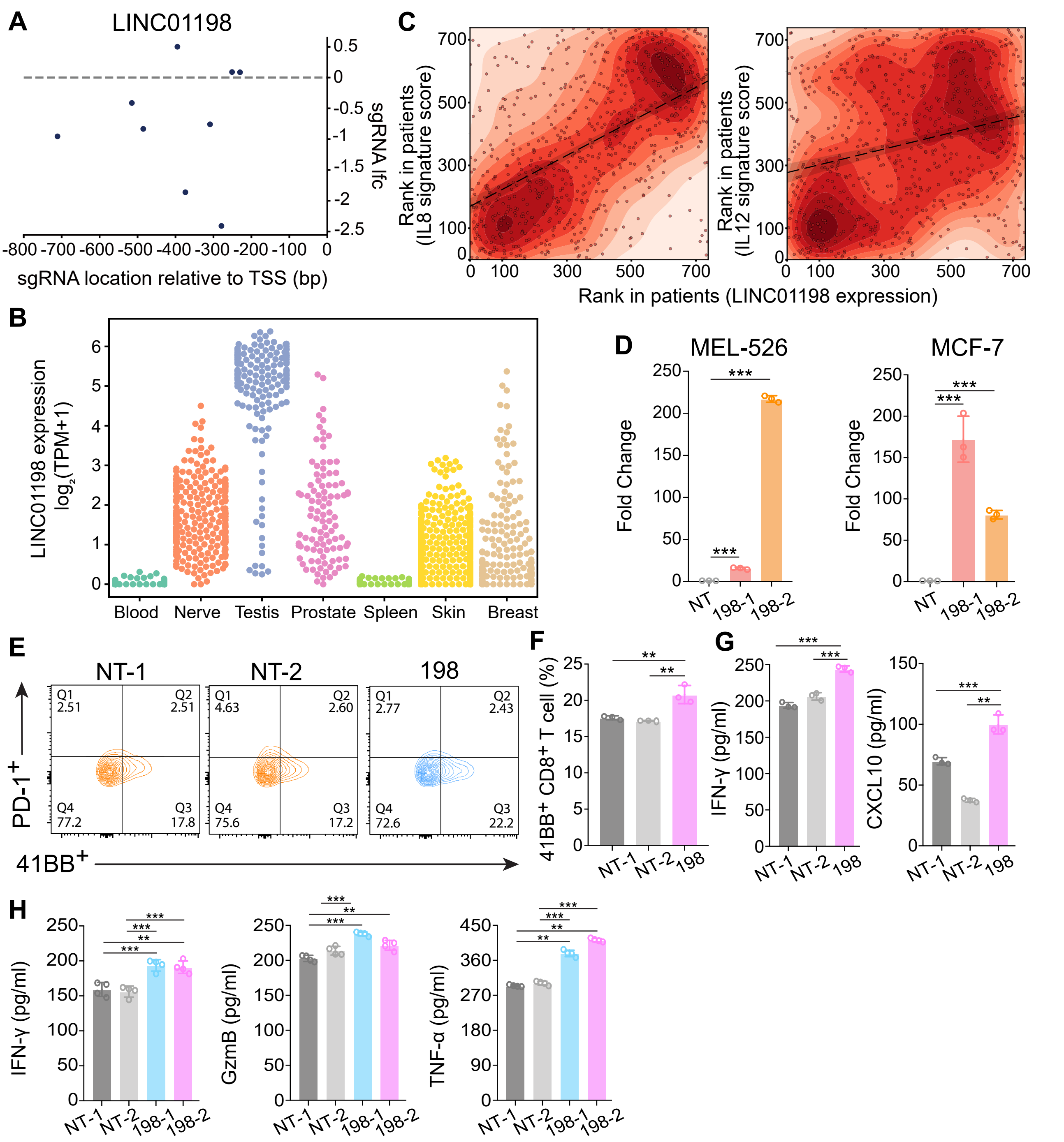

fig. S4. LncRNA *LINC01198* promotes CD8$^+$ T cell-mediated immune response in melanoma and breast cancer. (**A**) The location and log$_2$ fold change for 9 sgRNAs targeting *LINC01198*'s TSS. (**B**) *LINC01198*'s expression in seven normal tissues from the GTEx dataset. (**C**) *LINC01198*'s expression is positively correlated with IL8 (left) and IL12 (right) signature score in TCGA BRCA patients. (**D**) Real-time PCR result showing the mRNA expression level of *LINC01198* activation in MEL-526 (upper) and MCF-7 (lower) cells transfected with either non-targeting sgRNA (NT) control or single sgRNA activated *LINC01198* (198) (n = 3 independent experiment, mean ± s.d., two-tailed t-test, unpaired). (**E**) Representative FACS analysis panel of gp100 TCR-transduced CD8$^+$ T cells co-cultured with MEL-526 cells transfected with either non-targeting sgRNA (NT) control or single sgRNA activated *LINC01198* (198) with E:T ratio of 0.5:1 for 6 h. (**F**) Quantification of gp100 TCR-transduced CD8$^+$ T activation level of (E)**.** The data shown represent one of three independent experiments with similar results (mean ± s.d., two-tailed t-test, unpaired). (**G**) IFN-γ and CXCL10 secretion level from 6 h co-culture supernatant detected by ELISA. The data shown represent one of three independent experiments for three replicates of each group with similar results (mean ± s.d., two-tailed t-test, unpaired). (**H**) IFN-γ, Granzyme B (GzmB), TNF-α secretion level from 12 h co-culture supernatant detected by ELISA. The data shown represent one of three independent experiments with similar results(mean ± s.d., two-tailed t-test, unpaired). Statistical significance: *p < 0.05, **p < 0.01, ***p < 0.001.

figure S5

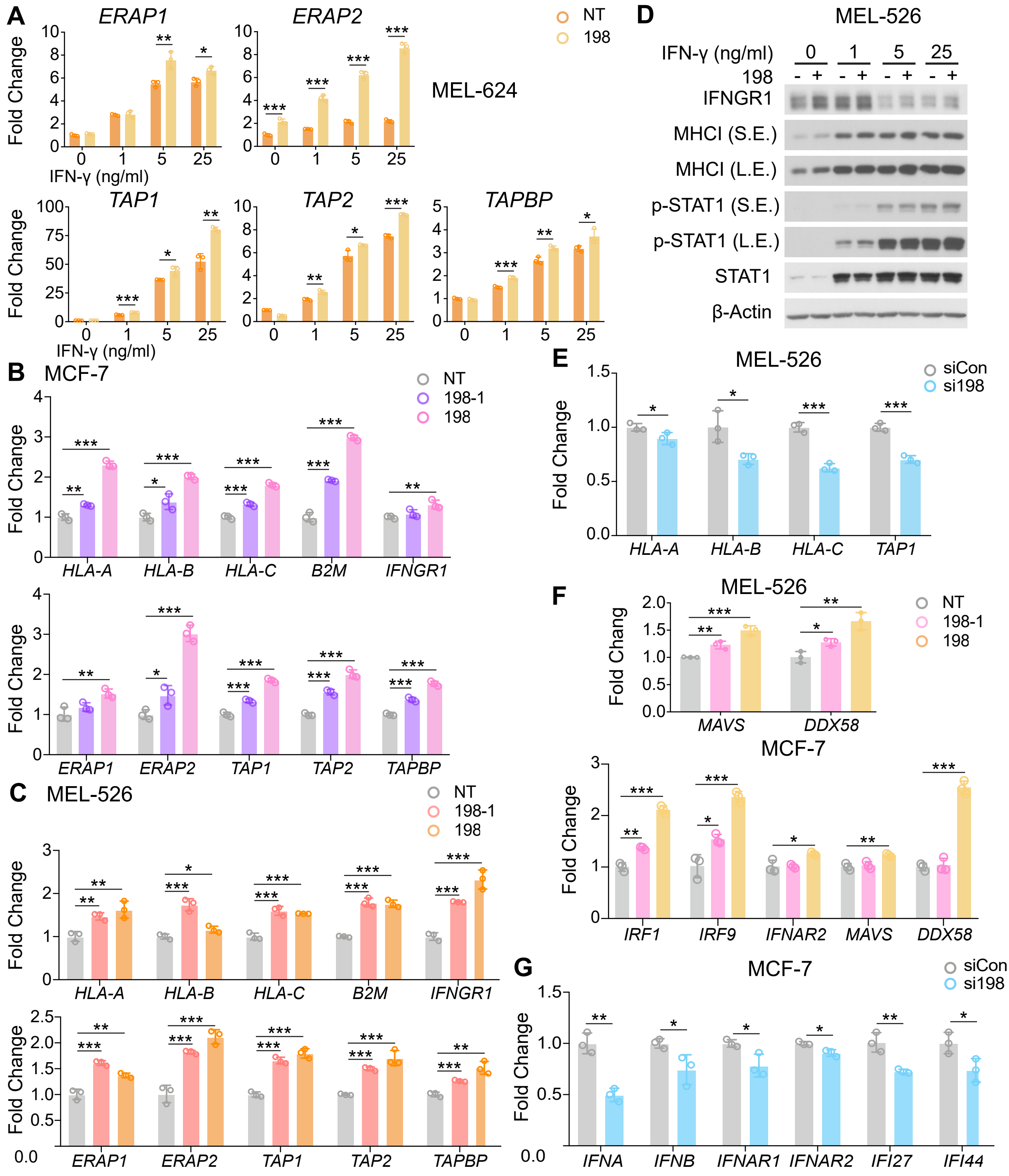

fig. S5. *LINC01198* regulates interferon signaling pathways. (**A**) Real-time PCR result showing the mRNA expression level of antigen processing genes in MEL-624 control (NT) and *LINC01198* activated (198) cells treated with IFN-γ (0, 1, 5, 25 ng/ml) for 24 h (n = 3 independent experiment, mean ± s.d., two-tailed t-test, unpaired). (**B, C**) Real-time PCR result showing the mRNA expression level of type II interferon-related genes in MCF-7 (B) and MEL-526 (C) control (NT) and *LINC01198* activated (198) cell (n = 3 independent experiment, mean ± s.d., two-tailed t-test, unpaired). (**D**) Representative western blot of IFNGR1, MHCI, p-STAT1 in MEL-526 control (- 198) and *LINC01198* activated (+ 198) cells treated with IFN-γ (0, 1, 5, 25 ng/ml) for 24 h. Data are representative of three independent experiments. Blots are cropped and original images can be found in the source data. (**E**) Real-time PCR result showing the mRNA expression level of type II interferon-related genes in MEL-526 *LINC01198* or control siRNA treated cells (n = 3 independent experiment, mean ± s.d., two-tailed t-test, unpaired). (**F** and **G**) Real-time PCR result showing the mRNA expression level of type I interferon-related genes of control (NT) and *LINC01198* activated (198) MEL-526 (upper) and MCF-7 (lower) cells (F), MCF-7 *LINC01198* or control siRNA treated cells (G) (n = 3 independent experiment, mean ± s.d., two-tailed t-test, unpaired). Statistical significance: *$p < 0.05$, **$p < 0.01$, ***$p < 0.001$.

figure S6

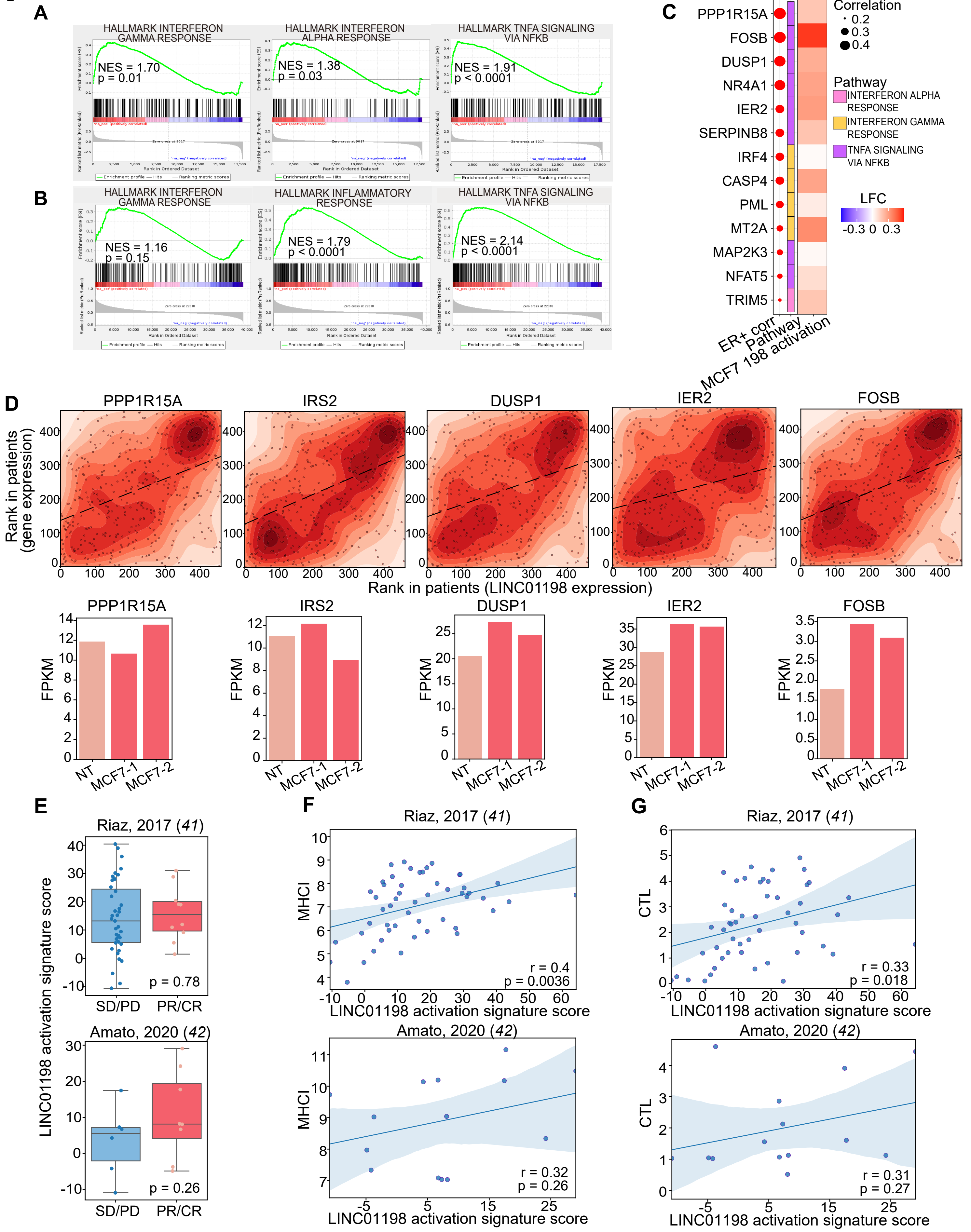
A
HALLMARK INTERFERON GAMMA RESPONSE
NES = 1.70
p = 0.01
HALLMARK INTERFERON ALPHA RESPONSE
NES = 1.38
p = 0.03
HALLMARK TNFA SIGNALING VIA NFKB
NES = 1.91
p < 0.0001
B
HALLMARK INTERFERON GAMMA RESPONSE
NES = 1.16
p = 0.15
HALLMARK INFLAMMATORY RESPONSE
NES = 1.79
p < 0.0001
HALLMARK TNFA SIGNALING VIA NFKB
NES = 2.14
p < 0.0001
Enrichment score (ES)
Ranked list metric (PreRanked)
Rank in Ordered Dataset
Enrichment profile — Hits
Ranking metric scores
C
PPP1R15A
FOSB
DUSP1
NR4A1
IER2
SERPINB8
IRF4
CASP4
PML
MT2A
MAP2K3
NFAT5
TRIM5
Correlation
0.2
0.3
0.4
Pathway
INTERFERON ALPHA RESPONSE
INTERFERON GAMMA RESPONSE
TNFA SIGNALING VIA NFKB
LFC
-0.3 0 0.3
ER+ corr
Pathway
MCF7 198 activation
D
PPP1R15A
IRS2
DUSP1
IER2
FOSB
Rank in patients (gene expression)
Rank in patients (LINC01198 expression)
FPKM
NT
MCF7-1
MCF7-2
E
Riaz, 2017 (41)
p = 0.78
SD/PD
PR/CR
Amato, 2020 (42)
p = 0.26
LINC01198 activation signature score
F
Riaz, 2017 (41)
MHCI
r = 0.4
p = 0.0036
LINC01198 activation signature score
Amato, 2020 (42)
r = 0.32
p = 0.26
G
Riaz, 2017 (41)
CTL
r = 0.33
p = 0.018
Amato, 2020 (42)
r = 0.31
p = 0.27

fig. S6. RNA-seq and signature analysis reveals *LINC01198*'s function in the patient studies and signaling pathways. (**A**) Immune-related gene sets were enriched with genes upregulated after *LINC01198* activation in MEL-526 cells. (**B**) Immune-related gene sets were enriched with genes positively correlated with *LINC01198*'s expression in TCGA ER+ breast cancer patients. (**C**) *LINC01198* activation significantly increased gene expression of immune-related pathways in MEL-526 cells. (**D**) *LINC01198*'s expression is positively correlated with five immune-related protein-coding genes in TCGA breast cancer patients (left) and *LINC01198* activation led to the five genes' over-expression in MCF-7 cells (right). (**E**-**G**) *LINC01198*-activation signature is positively correlated with anti-PD1 response (E), MHC-I expression (F), and cytotoxic T lymphocyte (CTL) infiltration (G) in two melanoma patient cohorts.

figure S7

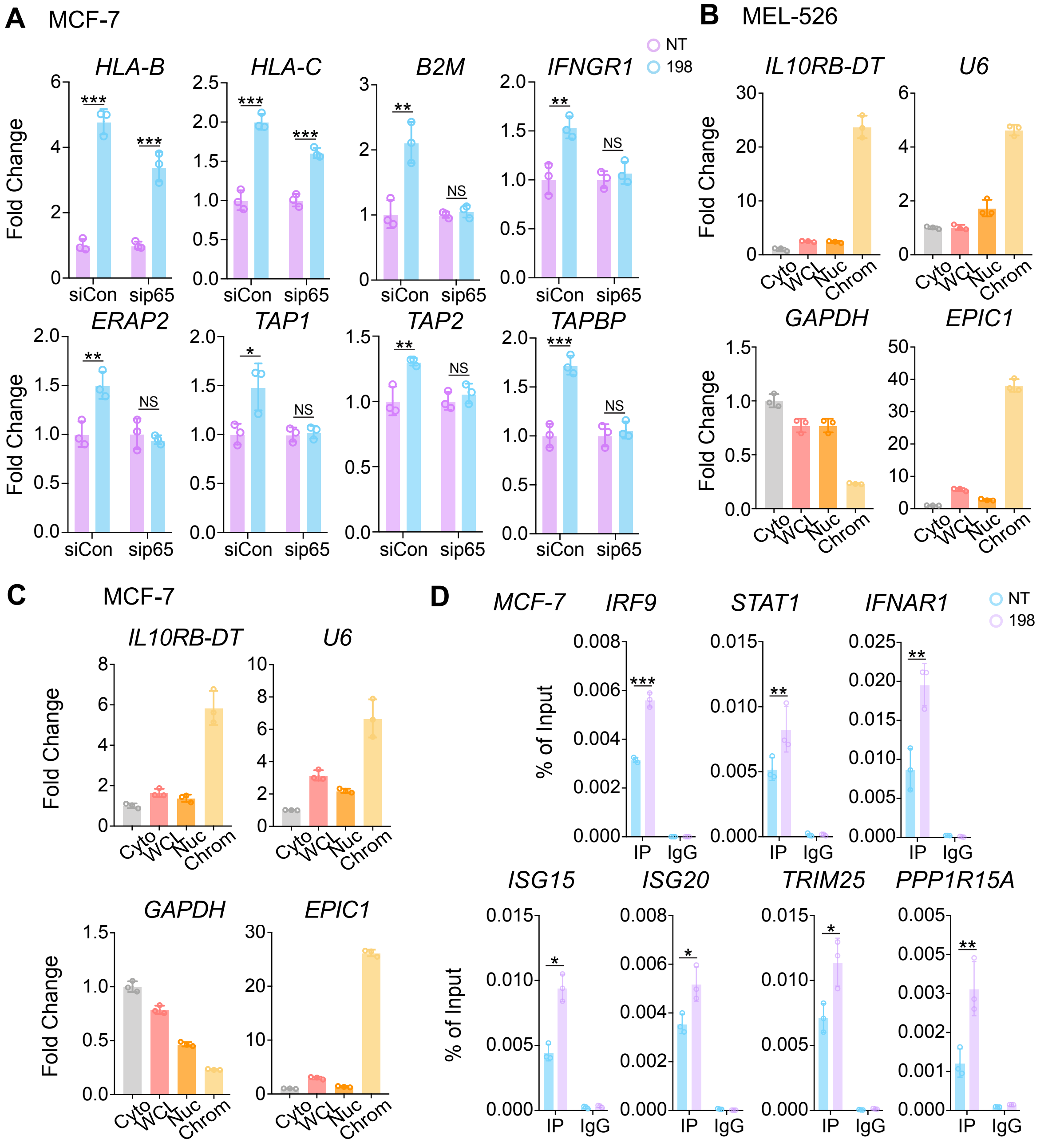

fig. S7. **Mechanism study indicates that *LINC01198* interacts and activates p65.** (**A**) Real-time PCR result showing the mRNA expression level of antigen presentation and processing genes in MCF-7 control (NT) and *LINC01198* activated (198) cells treated with either control or p65 siRNA. The expression level in *LINC01198* activated cells were normalized with its own control sample (n = 3 independent experiment, mean ± s.d., two-tailed t-test, unpaired). (**B** and **C**) Real-time PCR result of *IL10RB-DT*, *U6*, *GAPDH*, *EPIC1* expression of fractionation assay in MEL-526 (B) and MCF-7 (C) cells. *GAPDH*, *U6*, and *EPIC1* served as a specific cytoplasmic, chromatin marker in whole-cell lysates (WCL), cytoplasmic (Cyto), nuclear (Nuc), and chromatin (Chrom) fractionation. Data were normalized to the cytoplasmic fraction. Data shown represent one of three independent experiments with similar results (mean ± s.d., two-tailed t-test, unpaired). (**D**) ChIP-qPCR result of p65 occupancy on the promoters of its target genes in MCF-7 control (NT) and *LINC01198* activated (198) cells (n = 3 independent experiment, mean ± s.d., two-tailed t-test, unpaired). Statistical significance: *p < 0.05, **p < 0.01, ***p < 0.001. NS, not significant.

Other Supplementary Materials for this manuscript include the following:

Data S1. sgRNA distribution of CRISPRa screen.
Data S2. Analysis results of the CRISPRa screen for tumor response to T cell cytotoxicity.
Data S3. Differential expression and pathway analysis of lncRNA regulated genes.
